# SQANTI: extensive characterization of long-read transcript sequences for quality control in full-length transcriptome identification and quantification

**DOI:** 10.1101/gr.222976.117

**Published:** 2018-03

**Authors:** Manuel Tardaguila, Lorena de la Fuente, Cristina Marti, Cécile Pereira, Francisco Jose Pardo-Palacios, Hector del Risco, Marc Ferrell, Maravillas Mellado, Marissa Macchietto, Kenneth Verheggen, Mariola Edelmann, Iakes Ezkurdia, Jesus Vazquez, Michael Tress, Ali Mortazavi, Lennart Martens, Susana Rodriguez-Navarro, Victoria Moreno-Manzano, Ana Conesa

**Affiliations:** 1 Department of Microbiology and Cell Science, Institute for Food and Agricultural Sciences, Genetics Institute, University of Florida, Gainesville, Florida 32611, USA;; 2 Genomics of Gene Expression Laboratory, Centro de Investigaciones Principe Felipe (CIPF), 46012 Valencia, Spain;; 3 Neural Regeneration Laboratory, CIPF, 46012 Valencia, Spain;; 4 Department of Developmental and Cell Biology, University of California, Irvine, California 92617, USA;; 5 VIB-UGent Center for Medical Biotechnology, VIB, B-9000 Ghent, Belgium;; 6 Department of Biochemistry, Ghent University, B-9000 Ghent, Belgium;; 7 Centro Nacional de Investigaciones Cardiovasculares CNIC, 28029 Madrid, Spain;; 8 Spanish National Cancer Research Centre (CNIO), 28029 Madrid, Spain;; 9 Gene Expression and mRNA Metabolism Laboratory, CSIC, IBV, 46010 Valencia, Spain;; 10 Gene Expression and mRNA Metabolism Laboratory, CIPF, 46012 Valencia, Spain

## Abstract

High-throughput sequencing of full-length transcripts using long reads has paved the way for the discovery of thousands of novel transcripts, even in well-annotated mammalian species. The advances in sequencing technology have created a need for studies and tools that can characterize these novel variants. Here, we present SQANTI, an automated pipeline for the classification of long-read transcripts that can assess the quality of data and the preprocessing pipeline using 47 unique descriptors. We apply SQANTI to a neuronal mouse transcriptome using Pacific Biosciences (PacBio) long reads and illustrate how the tool is effective in characterizing and describing the composition of the full-length transcriptome. We perform extensive evaluation of ToFU PacBio transcripts by PCR to reveal that an important number of the novel transcripts are technical artifacts of the sequencing approach and that SQANTI quality descriptors can be used to engineer a filtering strategy to remove them. Most novel transcripts in this curated transcriptome are novel combinations of existing splice sites, resulting more frequently in novel ORFs than novel UTRs, and are enriched in both general metabolic and neural-specific functions. We show that these new transcripts have a major impact in the correct quantification of transcript levels by state-of-the-art short-read-based quantification algorithms. By comparing our iso-transcriptome with public proteomics databases, we find that alternative isoforms are elusive to proteogenomics detection. SQANTI allows the user to maximize the analytical outcome of long-read technologies by providing the tools to deliver quality-evaluated and curated full-length transcriptomes.

Alternative splicing (AS) and alternative polyadenylation (APA) are among the most fascinating and challenging aspects of eukaryotic transcriptomes. AS and APA are considered to be the major mechanisms of generating transcriptome complexity and thus the expansion of proteome diversity of higher organisms (Lu et al. 2010; Mudge et al. 2011; Frankish et al. 2012). These post-transcriptional mechanisms have been reported to play critical roles in differentiation (Wang et al. 2009; Martinez and Lynch 2013; Raj and Blencowe 2015; Teichroeb et al. 2016), speciation (McGuire et al. 2008; Mudge et al. 2011), and multiple human diseases such as cancer (Ladomery 2013; Liu and Cheng 2013; Chen and Weiss 2014), diabetes (Eizirik et al. 2012; Tang et al. 2015), and neurological disorders (Yang et al. 1998; D'Souza et al. 1999; Kanadia et al. 2003; Ladd 2013; Lee et al. 2016) and therefore may play a fundamental role in the establishment of organismal complexity (Black 2003; Mudge et al. 2011; La Cognata et al. 2014). The genome-wide analysis of AS has been done primarily using exon microarrays first and, more recently, short-read RNA-seq. These two methods are effective for the identification of AS events such as exon skipping or intron retention and have established the involvement of AS in many biological processes. However, both technologies have serious limitations for the reconstruction of the actual expressed transcripts, as short reads break the continuity of the transcript sequences and fail to resolve assembly ambiguities at complex loci (Steijger et al. 2013; Tilgner et al. 2014). This impairs any studies that would catalog specific transcriptomes, investigate *cis*-acting mechanisms within transcripts, infer open reading frames, or understand functional aspects of isoform diversity.

There has been increasing interest in the application of single-molecule sequencing to obtain full-length transcripts in animals and plants, as long reads allow direct sequencing and eliminate the need for short-read assembly and transcript reconstruction. Currently, there are three different long-read transcriptome sequencing platforms: Pacific Biosciences (PacBio) (Sharon et al. 2013; Tilgner et al. 2014; Li et al. 2016), Moleculo (Tilgner et al. 2015), and Nanopore (Oikonomopoulos et al. 2016). Here, we have used the popular PacBio Iso-Seq protocol, which consists of full-length cDNA enrichment using the Clontech SMARTer kit followed by building single-molecule SMRTbell libraries with specific PacBio linkers that are subsequently sequenced. PacBio reads are typically longer than the full-length cDNA sequence, which means that each molecule can go through several passes of sequencing. The consensus of these passes is called a Read of Insert (RoI), which is the current standard PacBio output. RoIs where both cDNA primers and the poly(A) can be identified are called Full-length (FL) reads, while those that miss any of these tags are deemed non-Full-length (non-FL) reads. PacBio sequencing suffers, however, from a relatively high raw error rate (∼15%) (Carneiro et al. 2012) and a lower throughput compared to Illumina. There are several described methods for PacBio error correction and transcript identification. Hybrid error correction methods such as LoRDEC (Salmela and Rivals 2014) and IDP (Au et al. 2013) were the first to appear. While LoRDEC corrects long sequences by traversing paths in de Bruijn graphs representing short-reads, IDP calls transcripts by using a combination of direct detection and prediction with short-reads that involves long-read correction by the computationally intensive LSC algorithm and genome alignment (Au et al. 2012). The TAPIS pipeline does not need Illumina reads but performs several rounds of mapping and correction of RoIs on the reference genome, with apparently similar error correction efficiency as a short-read-based method (Abdel-Ghany et al. 2016). Finally, the ToFU PacBio pipeline (Gordon et al. 2015) obtains auto-clusters of FL and non-FL RoIs and then computes a consensus transcript sequence where errors are significantly reduced. In all cases, comparison to the reference gene models serves to call known and novel transcripts.

All PacBio transcriptome papers discover thousands of new transcripts, propose classification schemes by comparing to a reference annotation, and find that the majority of novel transcripts appear in known genes (Au et al. 2013; Sharon et al. 2013; Tilgner et al. 2015; Abdel-Ghany et al. 2016; Wang et al. 2016). However, details on the number, quality, and characteristics of these new calls can vary greatly. Sequencing the transcriptome of hESCs by long reads followed by IDP analysis identified over 2000 novel transcripts (∼30%) and discovered new genes that were proven to be functional (Au et al. 2013). Tilgner et al. (2015) found about 12,000 novel transcripts fully supported by previous splice site annotations or Illumina reads using PacBio sequencing of the GM12878 cell line but did not study novel junctions in detail. For the sorghum transcriptome, 11,342 (40%) novel transcripts were found by PacBio from a total of nearly 1 million reads using a filter on splice junction quality (SpliceGrapher) (Rogers et al. 2012), and 6/6 random transcripts were confirmed by PCR. Finally, a maize multitissue transcriptome analysis identified over 111,151 transcripts from 3.7 million RoIs, most of them novel and tissue-specific (Wang et al. 2016). The authors found that between 10% and 20% of the PacBio junctions lacked coverage by Illumina reads and that <1% were noncanonical (Wang et al. 2016), but they did not report on the number of affected transcripts or carry out any validation. In all these cases, an in-depth characterization of the novel transcripts and junctions that would reveal potential biases and justify analysis choices was missing. We believe that such analysis is important, as a great variety of FL and non-FL RoIs typically map at each genome locus, and different processing pipelines can result in significantly different final transcript calls. As an example, sequencing the mouse neural transcriptome with PacBio, we obtained ∼90,000, 13,000, and 16,000 different transcripts when applying the TAPIS, IDP, or ToFU pipelines, respectively. Implementing a comprehensive, quality-aware analysis of single-molecule sequencing is fundamental at a time when long-read methods are becoming more popular and important conclusions on transcriptome diversity will be drawn from these data.

In this work, we present SQANTI (Structural and Quality Annotation of Novel Transcript Isoforms), a pipeline for the analysis of long-read transcriptomics data that creates a wide range of summary graphs to aid in the interpretation of the sequencing output, defines up to 47 different descriptors of transcript and junction properties, and uses these descriptors to implement a machine learning algorithm that removes artifactual transcripts.

## Results

### Overview of SQANTI analysis workflow

**Figure 1.**
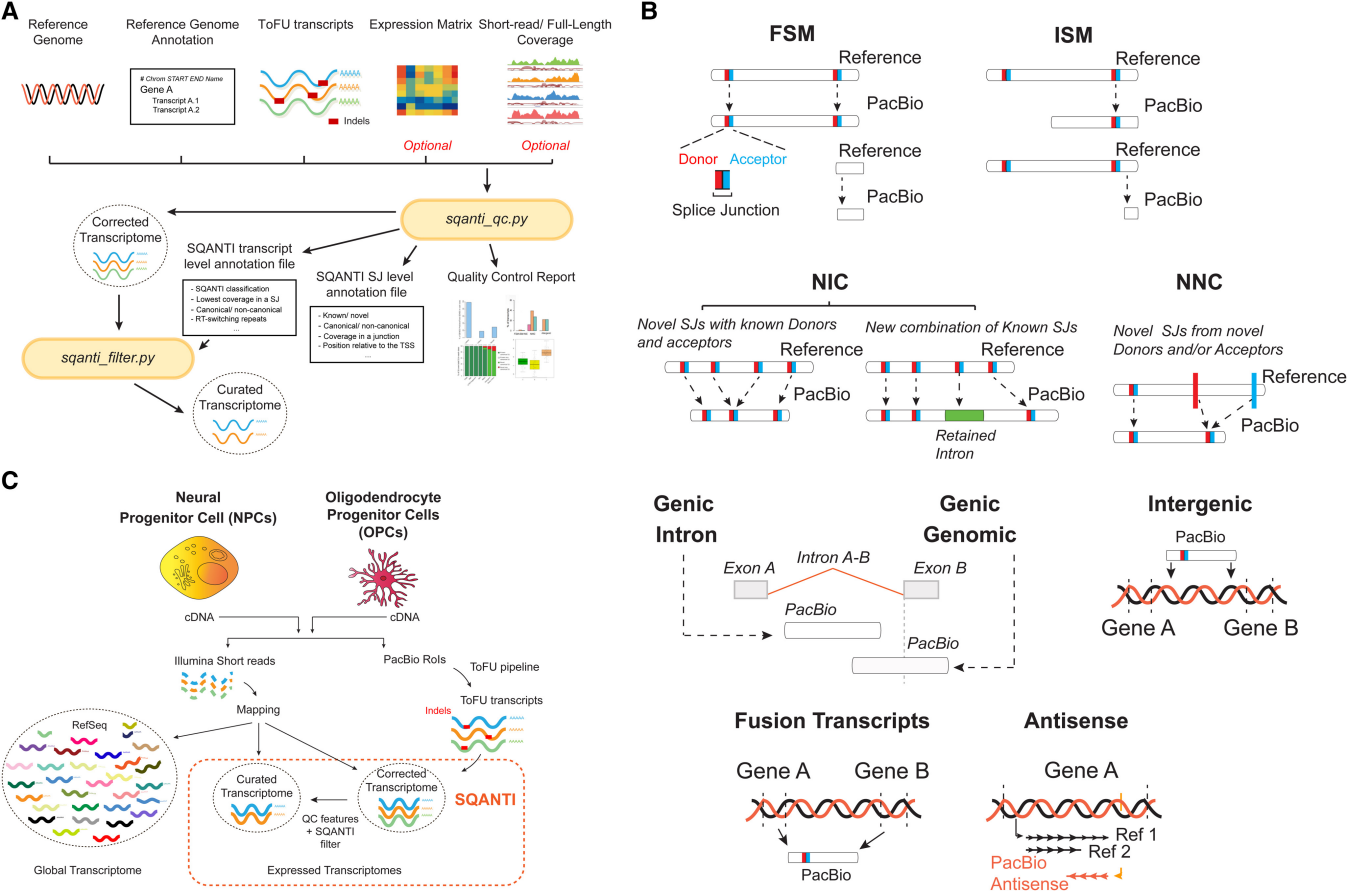
Overview of the experimental model and SQANTI analysis. (*A*) SQANTI workflow. Two main functions are part of SQANTI. *sqanti_qc.py* uses as input files a FASTA file with transcript sequences, the reference genome in FASTA format, a GTF annotation file, and optionally, full-length and short-read expression files. The function returns a reference-corrected transcriptome, transcript-level and junction-level files with structural and quality descriptors, and a QC graphical report. *sqanti_filter.py* takes the reference-corrected transcriptome and the transcript-level descriptors file to return a curated transcriptome from which artifacts have been removed. (*B*) SQANTI classification of transcripts according to their splice junctions and donor and acceptor sites. Splice donors and acceptors are indicated in red and blue, respectively. (SJ) splice junction, (FSM) Full Splice Match, (ISM) Incomplete Splice Match, (NIC) Novel in Catalog, (NNC) Novel Not in Catalog. (*C*) Experimental system and data processing pipeline. RNA isolated from neural progenitor cells (NPCs) and oligodendrocyte precursor cells (OPCs) was retrotranscribed separately into cDNA and sequenced both by long-read PacBio and short-read Illumina technologies. All PacBio RoIs were joined and processed by the ToFU pipeline to obtain consensus transcripts. Residual (indel) errors were eliminated by comparison to the reference genome to generate a corrected transcriptome, and false transcripts were removed using a SQANTI filter to result in a curated transcriptome. Illumina short reads were mapped against the RefSeq murine transcriptome annotation, the corrected, and the curated PacBio transcriptomes.

The SQANTI pipeline was developed for an in-depth characterization and curation of long-reads transcriptomes. SQANTI takes as input a transcripts data set, together with genome annotation and, if available, quantification data, to return a reference corrected transcriptome together with a wide set of transcript and junction descriptors which are further analyzed in several diagnostic plots (Fig. 1A). Supplemental Tables 1 and 2 describe in detail the set of descriptors computed by SQANTI at the transcript and junction levels, respectively. When necessary, the software can also filter out potential artifact transcripts using a SQANTI descriptor-based machine learning classifier.

#### Transcript and junction annotation

A feature of the SQANTI pipeline is that it can reveal the nature and magnitude of the novelty found by long-read sequencing by classifying transcripts based on the comparison between their splice junctions and the reference transcriptome provided (Fig. 1B). PacBio transcripts matching a reference transcript at all splice junctions are labeled as Full Splice Match (FSM), while transcripts matching consecutive, but not all, splice junctions of the reference transcripts are designated as Incomplete Splice Match (ISM). Those ISM transcripts with 95% or more of their sequence within the UTR3 sequence of their cognate reference transcript are labeled UTR3 Fragment. Monoexonic transcripts matching a monoexonic reference are included in the FSM category, whereas those matching a multiexonic reference are placed in the ISM group.

Furthermore, SQANTI classifies novel transcripts of known genes into two categories: Novel in Catalog (NIC) and Novel Not in Catalog (NNC). NIC transcripts contain new combinations of already annotated splice junctions or novel splice junctions formed from already annotated donors and acceptors. NNC transcripts use novel donors and/or acceptors. Note that this transcript classification scheme captures the intron-based definition described by Tilgner et al. (2013), but SQANTI goes a step further by describing and subclassifying the type of novelties introduced by transcripts not matching the splice pattern of annotated references. Transcripts in novel genes are classified as “Intergenic” if lying outside the boundaries of an annotated gene and as “Genic Intron” if lying entirely within the boundaries of an annotated intron. In addition, the “Genic Genomic” category encompasses transcripts with partial exon and intron/intergenic overlap in a known gene (Fig. 1B). Finally, SQANTI labels transcripts as Fusion (transcript spanning two annotated loci) and Antisense (poly[A] containing transcripts overlapping the complementary strand of an annotated transcript). Additionally, SQANTI annotates transcript length, number of exons, and, for the FSM class, length of the reference transcript and distance of transcript 3′/5′ ends to the reference transcript 3′/5′ ends.

SQANTI analyzes transcripts in relation to their splice junctions. Splice junctions can be divided into canonical and noncanonical according to the two pairs of dinucleotides present at the beginning and end of the introns encompassed by the junctions. The combination of GT at the beginning and AG at the end of the intron is found in 98.9% of all the introns in the human genome (Parada et al. 2014). We considered GT-AG as well as GC-AG and AT-AC as canonical splicing (altogether, found in more than 99.9% of all human introns [Cocquet et al. 2006; Parada et al. 2014]) and all the other possible combinations as noncanonical splicing. SQANTI also allows users to provide their own set of canonical junctions. Further, SQANTI subdivides splice junctions between known, if they are present in the reference, and novel, if they are not. When matching FL and short-read quantification data are provided, SQANTI will also quantify the number of supporting FL reads, transcript expression, and coverage of junctions by short-read data.

Two other important QC features calculated by SQANTI are reverse transcriptase (RT) template switching and off-priming. RT switching is an intrinsic property of RTs that allows them to jump within or across template positions without terminating DNA synthesis. Secondary structures in RNA templates have been shown to enhance RT switching activity (Cocquet et al. 2006; Houseley and Tollervey 2010) and to cause gaps during cDNA synthesis. These gaps are interpreted as splicing events, which, due to their nonsplicing origin, are enriched for noncanonical junctions (Cocquet et al. 2006; Houseley and Tollervey 2010). A hallmark of RT switching is the presence of a direct repeat between the upstream mRNA boundary of the noncanonical intron and the intron region adjacent to the downstream exon boundary (Cocquet et al. 2006). SQANTI incorporates an algorithm to locate these direct repeats. SQANTI also evaluates possible off-priming of the oligo(dT) in A-rich regions of the mRNA template. Annealing of the oligo(dT) primer used in the first-strand synthesis of the cDNA to non-poly(A) tail adenine stretches present in not yet discarded intron-lariats or (pre)-messenger RNAs results in false cDNA molecules (Nam et al. 2002; Spies et al. 2013). SQANTI investigates these events by calculating the percentage of adenines (A) within a window of nucleotides downstream from the genetic coordinates corresponding to transcripts’ 3′ ends.

Finally, SQANTI implements the GeneMarkS-T (GMST) algorithm (Tang et al. 2015) to predict ORFs from transcript sequences (Supplemental Methods and Supplemental Fig. 1C–E).

#### Diagnostic graphs

Supplemental Table 3 lists the set of diagnostics plots returned by SQANTI, which include distribution of transcript lengths, expression level, number of exons, position of junctions, full-lengthness, and different quality features such as the proportion of noncanonical junctions, RT switching evidence, and junction coverage by short reads. In addition, SQANTI provides most of these graphs with a transcript category breakdown in order to facilitate quality assessment of the transcriptome obtained by the single-molecule sequencing.

#### SQANTI filtering

After reference-guided error correction, artifacts might still present in the resulting transcriptome. SQANTI removes artifactual transcripts by applying a machine learning classifier based on SQANTI features and sets of true and artifact transcripts provided by the user or inferred by the application. The SQANTI filter also includes an option to discard transcripts flagged as intra-priming candidates. The resulting curated transcriptome can be checked again with the SQANTI QC function to verify improvement in quality parameters.

### Experimental design and transcriptome sequencing

SQANTI was evaluated on a mouse neural differentiation PacBio data set. Full-length cDNA from neural progenitor cells (NPCs) and oligodendrocyte progenitor cells (OPCs), two biological replicates each, was obtained and split to prepare Illumina and PacBio sequencing libraries (Fig. 1C). PacBio sequencing was performed according to the Iso-Seq protocol to generate around 0.6 million RoIs per sample for a total of 2.2 million RoIs. Illumina sequencing resulted in approximately 60 million reads per sample. All PacBio RoIs were joined and processed by the ToFU pipeline (Gordon et al. 2015) to obtain a total of 16,104 primary transcripts. Alignment of the ToFU transcripts against the mouse reference genome (GMAP, assembly mm10) (Wu and Watanabe 2005) showed an average percentage of coverage and identity above 99.8%, suggesting that the PacBio nominal high raw read sequencing error is corrected by the ToFU clustering approach, as reported (Gordon et al. 2015). However, small indels (average size ∼1.2 nt) were still detected in 56.2% of the transcripts. These small indels did not affect the overall long-read mappability, as long reads with and without indels had no significant differences in the GMAP quality of mapping and occurred with no particular sequence context bias (Supplemental Fig. 1A), which is in agreement with the random profile of PacBio sequencing errors (http://www.pacb.com/uncategorized/a-closer-look-at-accuracy-in-pacbio/; Loomis et al. 2013). We first attempted to correct indels with matching Illumina short reads using *proovread* (Hackl et al. 2014) and LSC (Au et al. 2012). Although the number of transcripts with at least one indel decreased to 16%, this was still unsatisfactory for ORF prediction. Instead, transcripts were corrected using the reference genome sequence (Fig. 1C). By virtue of this strategy, all indels were removed and we obtained the corrected PacBio transcriptome. This corrected PacBio transcriptome contained a total of 16,104 transcripts resulting from the expression of 7704 different genes. Following the SQANTI classification, transcripts mapping a known reference (FSM, ISM) accounted for 60% of the transcriptome, and novel transcripts of known genes (NIC, NNC) made up 35.6% of our sequences. Transcripts in novel genes (Intergenic and Genic Intron categories) represented about 2.3% of our data while transcripts in the Antisense and Fusion classes amounted to 1.1% and 0.3%, respectively (Supplemental Fig. 1B). We found 11,999 nonredundant ORFs within a total of 14,395 coding transcripts, while 1709 transcripts were predicted to be “ORF-less.” The great majority of FSM, ISM, NIC, and NNC transcripts were predicted to have ORFs (97%, 90%, 87.8%, and 92.8%, respectively), while the remaining categories were mostly noncoding.

### Descriptive analysis of transcriptome complexity and transcript full-length made easy by SQANTI

**Figure 2.**
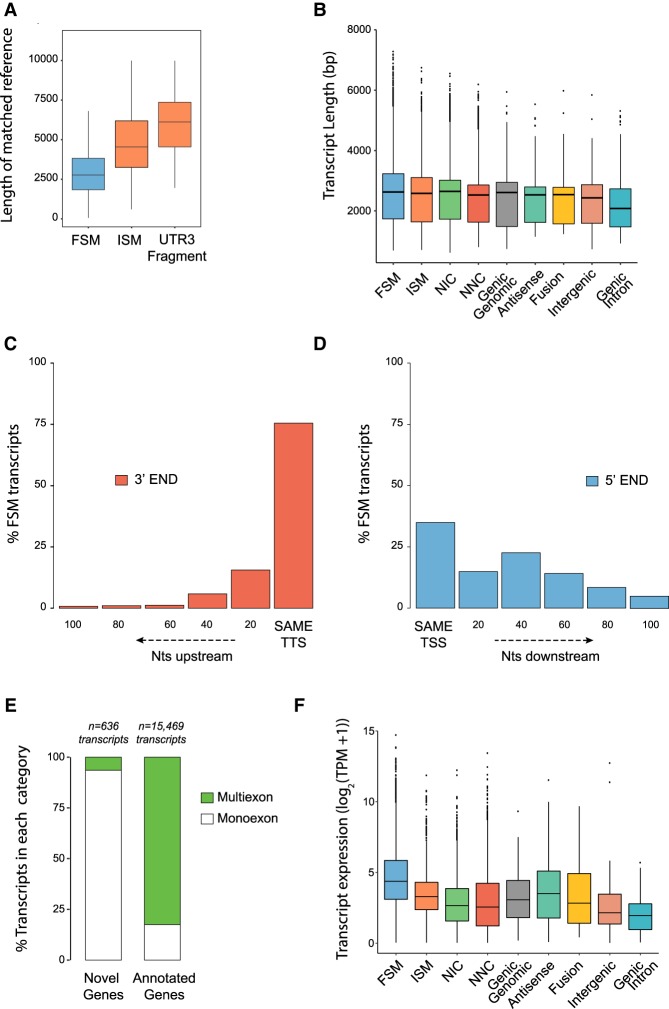
SQANTI characterization of the corrected PacBio transcriptome. (*A*) Length of the reference transcripts to which FSM, ISM, and UTR3 Fragment PacBio transcripts matched. (*B*) Length of PacBio transcripts by SQANTI categories. (*C*) Overlap at 3′ and (*D*) 5′ ends between the FSM transcripts and their respective matched reference transcripts. (TTS) transcription termination site, (TSS) transcription start site. (*E*) Percentage of monoexonic and multi-exonic transcripts for transcripts belonging to novel genes and annotated genes. (*F*) Transcript expression distribution across SQANTI categories.

A fundamental goal of long-read transcriptome sequencing is to capture the extent of transcriptome complexity and to obtain full-length transcripts. SQANTI includes all basic graphics to readily study these aspects. As analyses are provided with the transcript classification breakdown, this adds an extra layer of understanding to the quality of the sequencing results. For example, we hypothesized that ISM transcripts were a combination of potentially real shorter versions of long reference transcripts along with partial fragments resulting from incomplete retrotranscription or mRNA decay. Indeed, the SQANTI analysis showed that PacBio transcripts classified as ISM matched reference transcripts that were longer (Fig. 2A) and had more exons (Supplemental Fig. 2A) than FSM sequences. Moreover, UTR3 Fragment transcripts matched the longest reference transcripts (Fig. 2A), suggesting their enrichment in retrotranscription fragments. All transcript classes had similar median length (Fig. 2B), except for Genic Intron which was significantly lower (*t*-test *P*-value = 1.421 × 10^−15^), while this class and all novel gene categories except Fusion transcripts were almost entirely composed of monoexon transcripts (Supplemental Fig. 2B). In terms of full-lengthness, the majority of our FSM transcripts, as expected, showed a complete or close to complete 3′-end overlap with the 3′ end of the matched reference transcript: 76% had an exact 3′-end match and 16% were within 20 nt upstream of the annotated 3′ end (Fig. 2C). This contrasted with the 35% of FSM transcripts showing a complete overlap with their reference 5′ ends and 50% falling short by 40–100 nt (Fig. 2D). This result is in agreement with the strategy used in cDNA library preparation and ToFU analysis parameters that require identification of poly(A) tails to call FL reads but have less control over completeness at 5′ ends. Interestingly, 851 and 1361 FSM transcripts had 3′-end and 5′-end positions that extended beyond the matched reference transcript, while 1610 and 1439 of our FSM sequences were shorter by more than 100 nt at their 3′ and 5′ ends, respectively. These cases might represent alternative polyadenylation/alternative TSS events. Regarding novel genes, only 13.8% of them had splice junctions (Fig. 2E), and most (98.2%) expressed just one transcript (Supplemental Fig. 2C).

Finally, SQANTI descriptive graphs revealed differences in expression features between transcript categories at expression features. For example, transcript expression level and number of supporting FL reads were significantly lower for ISM, NIC, and NNC than for FSM transcripts (*t*-test *P* < 2.2 × 10^−16^ for all comparisons) (Fig. 2F; Supplemental Fig. 2D) and were significantly lower for novel genes compared to annotated genes (*t*-test *P* < 2.2 × 10^−16^ for both comparisons) (Supplemental Fig. 2E,F), which showed that novel transcripts had generally lower expression levels than those already identified in reference databases.

In summary, the descriptive analysis framework provided by SQANTI readily indicates that our neural mouse transcriptome, obtained by PacBio single-molecule sequencing, recovered full-length transcripts and had an important level of novelty (∼40%) with respect to the reference mouse transcriptome both because of novel splicing events and due to 3′-/5′-end length variation. Transcript diversity was more important than the presence of novel genes, which represented only a small fraction of the expressed mRNAs. However, novel transcripts tended to be less expressed than annotated transcripts, indicating that, generally, less novelty is to be expected for major transcripts.

### Evaluation of transcripts according to their splice junctions

**Figure 3.**
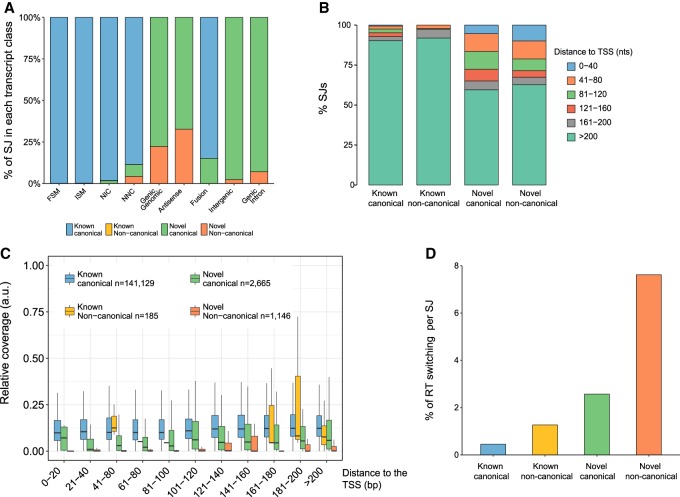
Splice junction characterization in the corrected PacBio transcriptome. (*A*) Distribution of splice junction (SJ) types across SQANTI categories. NNC, Genic Genomic, Antisense, Intergenic, and Genic Intron are enriched in noncanonical SJs. *n* = 76,757 SJ for FSM, *n* = 13,802 for ISM, *n* = 27,368 for NIC, *n* = 26,509 for NNC, *n* = 51 for Genic Genomic, *n* = 49 for Antisense, *n* = 494 for Fusion, *n* = 86 for Intergenic, and *n* = 55 for Genic Intron. (*B*) Distribution of the SJs according to their distance to the transcription start site. (*C*) Relative coverage by short reads of SJs as a function of their class and distance to the TSS. (a.u.) Arbitrary units. (*D*) Detection of RT switching direct repetitions by SQANTI algorithm across SJ types.

In our mouse neural data, the ratio of canonical versus noncanonical splicing events fitted the expected genome proportions when looking at known splice junctions: Out of 141,332 known splice junctions, 99.9% were canonical and 0.1% (185) were noncanonical. However, novel splice junctions showed a very different distribution: Out of 3837 novel splice junctions, 69% were canonical and 31% (1188) were noncanonical. When analyzed across the different SQANTI categories, noncanonical splicing was maintained at low rates in FSM (0.1%) and ISM (0.25%) transcripts, which was expected as both are formed purely by known splicing events (Fig. 3A). In NIC transcripts, made up of novel combinations of known splice junctions or novel splice junctions deriving from annotated donors or acceptors, the percentage of noncanonical splicing was 0.15% (Fig. 3A). In all cases, these noncanonical junctions were already known in the reference, and consequently all novel junctions found in this transcript category were canonical. However, in NNC transcripts, characterized by the introduction of alternative donors and/or acceptors, we found 1155 novel noncanonical junctions, which represented 4.5% of the total. Moreover, Genic Genomic, Intergenic, Genic Intron, and Antisense transcripts, despite rarely being multiexonic, showed relatively high percentages of noncanonical splice junctions, with 2.32%, 7.28%, 21.57%, and 32.65%, respectively (Fig. 3A). This unusually high level of noncanonical junctions suggests that experimental artifacts might be accumulating in these categories. Furthermore, when the percentage of transcripts showing at least one noncanonical splice junction was considered, the proportion of affected NNC compared to NIC transcripts became more evident, 41.5% vs. 1.47%, respectively (Fisher's exact test [FET] *P* < 2.2 × 10^−16^), strongly indicating that this category of transcripts needed deeper inspection.

We found, that although novel junctions could appear at any position in novel transcripts, there was a higher concentration of occurrences toward 5′ ends which is not observed for known—whether canonical or not—junctions (FET *P* < 2.2 × 10^−16^) (Fig. 3B). This could either be the consequence of unannotated variability at 5′ ends or higher accumulation of errors due to lower sequence support. The ToFU pipeline is more permissive with clustering conditions at transcript ends (E Tseng, pers. comm.), which accounts for a higher probability of errors at these areas.

Coverage by Illumina has been used to support novel junctions called by PacBio (Au et al. 2013). However, Illumina reads are not always equally distributed along the transcript length and are often less abundant toward 5′ ends, providing less support for junction validation. We found that, as suspected, splice junction support by short reads decreased toward the 5′ end of the transcripts but was significantly higher for known junctions (Wilcoxon rank-sum test [WRS] *P* < 2.2 × 10^−16^) (Fig. 3C). Novel canonical junctions were in general less frequently covered but still significantly more supported than novel noncanonical junctions, which had hardly any supporting reads if located within the first 120 nt of the transcript 5′ end (WRS *P* < 2.2 × 10^−16^) (Fig. 3C).

Another possible explanation for noncanonical splicing is RT switching. SQANTI analysis confirmed the enrichment of RT switching in novel splice junctions (FET *P* < 2.2 × 10^−16^) (Fig. 3D) and in NNC compared to NIC transcripts (7.24% versus 1.98%; FET *P* < 2.2 × 10^−16^). Described RT switching events affect minor isoforms of genes co-expressed with a major isoform that serves as the template for the intra-molecular switching (Cocquet et al. 2006). Accordingly, we found that NNC transcripts are enriched for being minor transcripts of highly expressed genes (Supplemental Fig. 2G,H). Finally, A-rich genomic DNA regions downstream from the TTS were concentrated in the relatively minor SQANTI categories (Supplemental Fig. 2I) and were enriched in noncoding and monoexonic transcripts (WRS *P* < 2.2 × 10^−16^ for all tests) (Supplemental Fig. 2J). A total of 601 transcripts were found to be intra-priming candidates, which affected the Antisense and Genic Intron categories in particular (∼50% and ∼ 30% of their transcripts were flagged). Remarkably, Incomplete Splice Match transcripts that were versions of the reference transcripts shortened at the 3′ end and monoexon NIC transcripts with intron retention events were also significantly enriched in intra-priming candidates (WRS *P* < 2.2 × 10^−16^ for all tests) (Supplemental Fig. 2I).

Altogether, the SQANTI framework analyses suggest that a fraction of the novel transcripts found by the ToFU pipeline could be technical artifacts that originated at the cDNA library construction step or via less confident sequencing data at the 5′ ends of transcripts.

### PCR validation of PacBio transcripts

To shed light on whether the transcripts detected by the ToFU analysis were correct or not, we performed RT-PCR amplifications for a total of 67 mRNAs encompassing different SQANTI categories: 23 FSM (three with noncanonical splice sites), 12 NIC, 30 NNC canonical (11 of them containing at least one noncanonical splice junction), and three Fusion (Supplemental Fig. 3). Importantly, we performed RT-PCRs both on the Clontech oligo(dT)-enriched full-length cDNAs used for PacBio sequencing and, for positive NIC/NNC/Fusion and four FSM transcripts, on new cDNA retrotranscribed at 42°C and 50°C using random hexamers rather than oligo(dT). The rationale behind this approach was to test whether novel transcripts could have been spuriously generated by RT switching-like mechanisms at the retrotranscription step of the PacBio protocol. Since higher temperature and/or the use of random hexamers would complicate the formation of secondary structures in the RNA template, retrotranscription artifacts would be less favored in these conditions.

**Figure 4.**
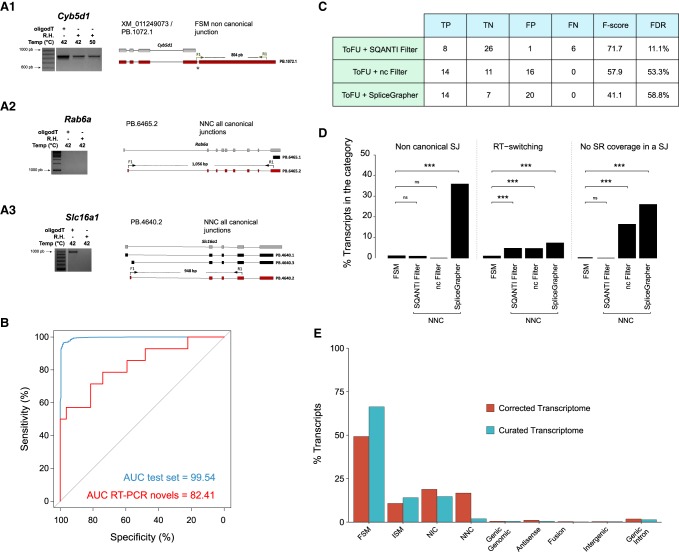
SQANTI filter performance on mouse data set. (*A*) Representative examples of RT-PCR validation experiments. Three PCR conditions were assessed: oligo(dT) template at 42°C and random hexamers (RH) template at 42°C and at 50°C. (*A1*) Example of a FSM transcript with a noncanonical SJ successfully amplified at each PCR condition. (*A2*) Example of a NNC transcript with a noncanonical SJ that failed to be amplified in the oligo(dT) condition. (*A3*) Example of NNC transcript with noncanonical SJ amplified at oligo(dT) but not at RH conditions. (*B*) ROC curves of the SQANTI ML filter for the test set (blue line) and for the set of novel isoforms assayed by RT-PCRs (red line). (*C*) Summary of the performances of the SQANTI filter, the noncanonical filter, and the SpliceGrapher filter for the set of novel isoforms assayed by RT-PCR. (nc filter) Noncanonical filter, (TP) True Positive, (TN) True Negative, (FP) False Positive, (FN) False Negative, (FDR) False Discovery Rate. (*D*) Comparison of quality features in the FSM and NNC categories after the SQANTI, nc, and SpliceGrapher filters. Statistical differences by Fisher's exact test (FET), (***) *P* < 0.001, (ns) not significant. (*E*) Composition of SQANTI transcript categories in the mouse before and after the SQANTI filter.

We validated by RT-PCR for all of the 23 FSM, including the three cases with noncanonical junctions (Fig. 4A1), highlighting the high level of confidence supporting these transcripts. Novel transcripts showed lower validation rates: 8/12 NIC, 1/3 Fusion, and 6/30 NNC, highlighting the low detection rate within the NNC category (Fig. 4A2). Importantly, nine of these nonvalidated NNC transcripts were amplified by oligo(dT) PCR but were lost when random hexamers and higher temperatures were used (Fig. 4A3), suggesting the possible occurrence of retrotranscription artifacts. Table 1 summarizes the results of the PCR validation experiment. Details can be found in Supplemental Table 4. These results indicated that an additional filtering strategy was important to remove artifactual transcripts from the ToFU transcriptome output.

**Table 1.**
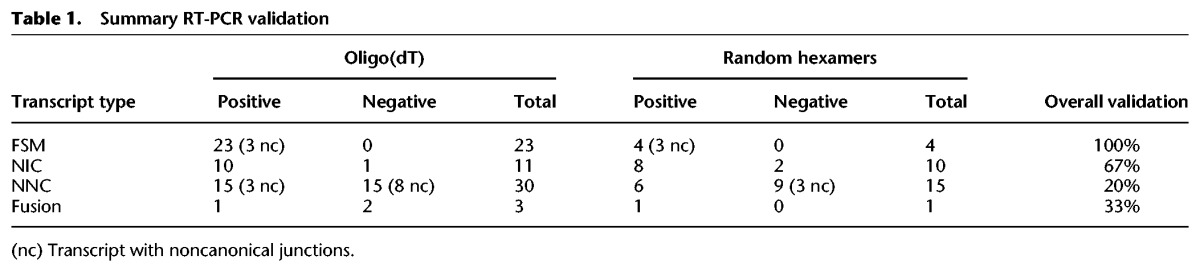
Summary RT-PCR validation

### Using SQANTI features to build a quality control filter for ToFU artifacts

Previous work applied different criteria to discard artifacts from transcriptome sequencing, including support by short reads (Au et al. 2012), removal of transcripts with noncanonical splicing (Tilgner et al. 2013), or filtering based on sequence features (Rogers et al. 2012). However, we found that these approaches do not fully capture the complexity of the data. For example, a few known and NIC transcript junctions lack Illumina coverage (148 out of 67,610, and 20 out of 437, respectively), while most of the novel noncanonical junctions did have supporting Illumina reads (543 out of 597). We found that additional features such as RT switching direct repeats and low expression values accumulated in NNC transcripts but were not exclusive to them. Moreover, our RT-PCR analysis revealed an important number of transcripts (16) having a full set of canonical junctions but failing validation.

We hypothesized that the set of SQANTI descriptors ought to be informative of transcript quality and could be used to define a composite filter to remove artifact transcripts efficiently. We decided to train a machine learning (ML) classifier based on these features. To obtain a generally applicable filter, we trained our classifier with a “best guess” of true (positive set) and artifact (negative set) transcripts within the genome-corrected ToFU output: We defined as the positive set Full Splice Match transcripts (*n* = 7774) and as the negative set Novel Not in Catalog transcripts with at least one noncanonical junction (NNC-NC; *n* = 1110). Note we trained the classifier without providing this structural information (Methods). We used Random Forest (Breiman 2001) with an 80/20 training/test set split, random down-sampling for class balance, and 10× cross-validation, and called predicted transcripts those with a probability for positive classification higher than 0.75. As a note, the RT-PCR instances mentioned in the previous section were excluded from the classifier build. We obtained an area under the curve (AUC) of 99.54% for the ROC curve of the test set (Fig. 4B, blue line), while the AUC for the set of NIC/NNC transcripts assayed by RT-PCR was 82.41% (Fig. 4B, red line, and Supplemental Table 5). This result indicates that our classifier built on SQANTI descriptors faithfully captures differences between our ground truth set of positive and negative transcripts, and this can be efficiently applied to discriminate true transcripts from artifacts within the set of long-read defined novel sequences. Figure 4C shows how well the RT-PCR data of the SQANTI classifier performs against two previous approaches used to remove artifacts, namely the “noncanonical splice junction” filter and SpliceGrapher. Data indicate that the classifier approach has a higher F1 score (71.7 versus 57.9 and 41.1, respectively) and lower FDR (11% versus 53.3% and 58.8%, respectively) than alternative methods. These notable FDR differences are mostly due to a high rate of false canonical junction transcripts that are not discarded by the prior approaches. Moreover, SQANTI was the only filtering strategy that succeeded in lowering both the noncanonical SJ and the no short-read coverage quality features in NNC transcripts to levels similar to those of the high-confidence FSM category (Fig. 4D).

Features selected by the SQANTI classifier are shown in order of importance in Supplemental Figure 4. The feature ranked first in order of importance (Bite) flags transcripts that skip consecutive exons and have donor/acceptor sites inside a known exon, which we interpret as an indication of novel splice junctions caused by secondary RNA structures. Five out of the eight top variables (lowest Illumina coverage at junction, minimum sample coverage, number of FL reads, expression of the gene, expression of the transcript, and ratio of transcript versus gene expression) were associated with transcript expression, suggesting that expression patterns are within the most useful characteristics for calling bona fide novel transcripts.

Following these results, we incorporated a function for transcriptome curation into SQANTI. When applied to the mouse neural transcriptome, the combination of the SQANTI ML and intra-priming filters removed 4134 novel transcripts (2462 NNC, 1281 NIC, 32 Genic Genomic, 36 Fusion, 116 Antisense, 25 Intergenic, 129 Genic Intron, and 53 ISM). In our final curated transcriptome, the adjusted percentages of each category were: 66.3% FSM, 14.1% ISM, 15.7% NIC, 2% NNC, 0.5% Genic Genomic, 0.5% Antisense, 0.2% Fusion, 0.3% Intergenic, and 1.4% Genic Intron (Fig. 4E). The transcript category in which our filter has the strongest impact is NNC, that went from 14% to 2%, while FSM increased consequently from 49% to 66% in the curated transcriptome (Fig. 4E). In our final data set, 9626 transcripts (80.4%) are in the known categories, 2344 (19.6%) are novel transcripts, of which 207 (1.7%) fall within novel genes. These transcripts were the product of 7167 genes and resulted in 9269 different ORFs.

### Generalization of the SQANTI approach

To assess the general utility of SQANTI, we applied our approach to alternative analysis pipelines and data sets. We processed our raw mouse PacBio reads with the IDP and TAPIS pipelines and analyzed resulting transcriptomes with SQANTI (Supplemental Fig. 5A,B). IDP, which relies heavily on a high-quality reference annotation and on short-reads correction, returned a total of 13,525 transcripts, the great majority belonging to the FSM category (96%). Only 509 transcripts were novel in this approach (358 NIC, 158 NNC), yet they still showed significant enrichments in RT switching and no short-read coverage in a junction (Supplemental Fig. 5A). IDP fails to return any of the 16 novel transcripts validated by PCR, suggesting that this method is highly restrictive for novel isoform calling. On the contrary TAPIS, that, like ToFU, works without short-read data, returned a significantly larger set of transcripts (91,428), with an overwhelming majority of them belonging to the NNC class (66%), which were strongly enriched in low-quality features (Supplemental Fig. 5B).

We next evaluated our analysis pipeline using additional data sets, namely the maize ear (Wang et al. 2016) and the human MCF-7 cells (http://www.pacb.com/blog/data-release-human-mcf-7-transcriptome/), both publicly available. Transcriptome composition in these data sets was substantially similar to what we observed for our mouse transcriptome, with a significant number of novel transcripts in known genes that were enriched in low-quality features (Supplemental Fig. 5C,D). We applied the SQANTI filtering approach to these data sets by training our ML classifier in each case with their sets of FSM and NNC-NC transcripts and using default values for removing of intra-priming events. As with the mouse data, we obtained high AUC values in the test sets (99.3% for maize ear and 99.7% for MCF-7) and succeeded in removing a considerable amount of low-quality novel transcripts while controlling their enrichment in low-quality features (Supplemental Fig. 5C,D).

Additionally, we analyzed the importance of SQANTI descriptors for the ML classifier in these data sets with respect to the mouse data. Although we observed an overall agreement in top-ranked classification features (i.e., the top three variables were shared among data sets), we also found some noticeable differences (Supplemental Fig. 4). For example, the number of FL reads was not a highly ranked feature for the maize ear data, probably due to the lower sequencing depth of this data set, and was absent for the MCF-7 data set, as the value was not available. Still, in both cases, the SQANTI classifier achieved high classification performance. We conclude that our SQANTI filtering approach based on the composite utilization of quality descriptors is a robust but versatile approach for effectively removing artifacts in long-read transcriptome data sets that can be applied to a wide range of organisms.

Altogether, this section shows that the SQANTI quality control framework is a very useful tool to reveal the structural composition of transcriptomes obtained from long-read sequencing and to compare quality across preprocessing pipelines and experiments. We show that our choice of ToFU read clustering plus SQANTI filtering for transcriptome curation is a good trade-off between discovery and high quality for novel transcript calls and can be efficiently applied to different PacBio long-read data sets provided that a reference genome and short-read data are available.

### Functional insights from novel and alternative transcripts

**Figure 5.**
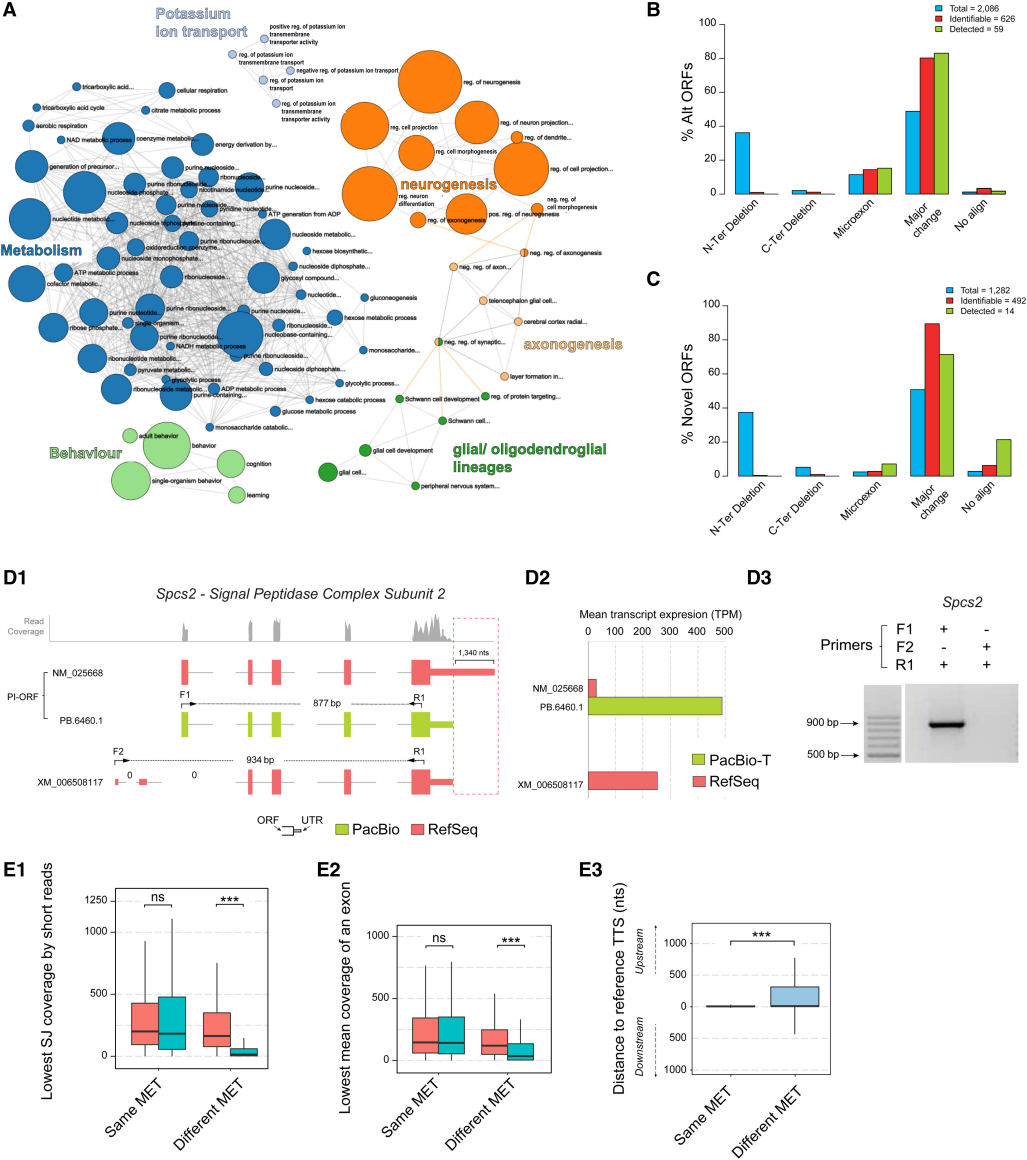
Functional diversity associated with genes with novel transcripts, variability of 3′ UTR in PI-ORFs transcripts, and comparative analysis of protein differences between PI and non-PI-ORFs. (*A*) Gene Ontology enrichment analysis for genes expressing novel transcripts. Analysis of the type of protein changes introduced by (*B*) Alternative ORFs and (*C*) Novel ORFs with respect to the PI-ORF of the gene. Blue: ORFs computationally predicted in the curated transcriptome; red: ORFs predicted to be identifiable by unique peptides; green: ORFs detected in proteomics databases with at least two peptide spectrum matches (PSMs). (*D*) Example of 3′ UTR variability in a PI-ORF that leads to a quantification error. (*D1*) Transcripts associated with the *Spcs2* gene according to PacBio sequencing (green) and by RSEM quantification using RefSeq (red). The profile of mapping short reads at the *Spcs2* locus is shown in gray. The positions of transcript-specific primers are indicated by arrows, and differences at the transcription termination sites are highlighted by a red dashed box; 0 indicates splice junctions lacking any short-read support. (*D2*) Short-reads-based average transcript expression levels of *Spcs2* transcripts using either RefSeq or PacBio-T references. (*D3*) Validation of *Spcs2* transcript expression by RT-PCR: PB.6460.1/ NM_025668 but not XM_006508117 was amplified. (*E*) Analysis of the most expressed transcript (MET) in genes with MET differences between PacBio-T and RefSeq quantifications. Kruskal-Wallis Test, (K-W), (***) *P* < 0.001, (ns) not significant. (*E1*) Lowest SJ coverage by short reads in METs. (*E2*) Lowest mean exon coverage by short reads in METs. (*E3*) Distance between the TTS of the METs and their FSM references. Same MET means both PacBio-T and RefSeq select the same MET; Different MET means RefSeq selects a MET that is not manually curated and PacBio-T selects a MET that is manually curated.

Most of the novel transcripts from the mouse neural transcriptome belong to existing genes. To further understand the biological relevance of these new calls, we analyzed the cellular processes where they participate. Genes with novel transcripts were enriched in metabolic processes, regulation of neurogenesis, oligodendroglial lineage, behavior, and regulation of potassium ion transport (Fig. 5A), suggesting that unannotated isoform diversity may impact fundamental energy utilization and specific neural biology pathways, both key for neural differentiation (Cai et al. 2004; Amaral et al. 2016; Shih et al. 2017). The availability of a full-length corrected and curated transcriptome allows us to predict ORFs with high confidence and annotate 3′ and 5′ UTRs. We studied to what extent alternative splicing modifies coding and noncoding regions of transcripts and impacts the principal isoform (PI) of the gene. PIs are defined by the APPRIS (Rodriguez et al. 2013) database as the protein isoform with highest functional load and cross-species conservation. Approximately, 36% of the genes expressed in our system were multi-isoform genes. Of these, 1836 genes expressed the transcript corresponding to the principal isoform (Rodriguez et al. 2013) of the gene and in 592 cases (32%), the PI transcripts were expressed with multiple, distinct UTR regions. Transcripts corresponding to predicted alternative ORFs were expressed for 1429 genes (79%). In contrast, these non-PI transcripts were much less variable at UTRs, with only 9% of them showing multiple 3′ or 5′ UTR variants, and about 27% of the novel transcripts extended existing TSSs or TTSs. This result suggests that, at least in the mouse neural transcriptome, multi-isoform expression would mostly result in a change in the predicted protein and to a lesser extent in the alternative processing of UTRs. However, alternative ORFs rarely were expressed as more than one transcript, suggesting further transcriptional regulation of these alternative forms might not be required to modulate their functionality.

### Peptide support of novel and alternative transcripts

As most of the novel transcripts were predicted to have ORFs that contained novel amino acid stretches when compared to PIs, we sought to investigate whether peptide data present in public proteomics databases could support these findings. In order to do this, we first created a nonredundant ORF database of public mouse proteins and the predicted proteins in our neural data and classified each protein as a Principal Isoform ORF (PI-ORF; *n* = 4579) if annotated as such by APPRIS (Rodriguez et al. 2013), Alternative ORF (Alt-ORF; *n* = 2127) if present in Ensembl or RefSeq but not being PI, and Novel ORF (Novel-ORF; *n* = 1194), if the protein would be coded by NIC or NNC transcripts present only in our mouse PacBio data. For each predicted protein, we performed an in silico trypsin digestion and selected unique peptides that would unequivocally identify each ORF. We analyzed theoretical peptides for those genes identified in our mouse transcriptome that had more than one isoform annotated in Ensembl (v80). The percentage of ORFs predicted to be identifiable by unique peptides was highest for the PI-ORFs (56.3%, or 2577), followed by the Novel-ORFs (42.6%, or 509) and was lowest for Alt-ORFs (30.1%, or 641). The majority of Novel-ORFs and Alt-ORFs were predicted to have only one unique peptide, while this was only the case for 14.2% of the PI-ORFs (Supplemental Fig. 6A). Conversely, most PI-ORFs were predicted to contain six or more discriminating peptides and this was true for only 7% of Alt-ORFs and 9.8% of Novel-ORFs. This higher rate of unique peptides in PI-ORFs was expected as the mouse genome contains a significant number of genes in which alternative isoforms are incompletely annotated and have only partial sequences and the APPRIS PI is often the longest ORF in a gene. Consequently, proteins deemed as PIs are expected to be easier to detect by protein digestion approaches than alternative isoforms.

We then screened public databases for the presence of unique peptides associated with our set of ORFs. Two separate approaches were conducted: a Neural tissue approach, comprising one proteomics study of mouse neural tissue and another study of the mouse neural secretome, and an All tissue approach comprising peptides from 36 proteomics studies carried out on a variety of murine tissues but excluding the two used in the first approach. Overall, we detected at least one unique peptide for 77.9% of the PI-ORFs predicted to be identifiable, while this percentage went down to 20.56% and 8% for Alt-ORFs and Novel-ORFs, respectively. Most Alt- and Novel-ORFs had single unique peptide matches, while most PI-ORFs were found with multiple peptides (Supplemental Fig. 6B). In part, this is to be expected; the success of detection was significantly lower when the ORF was predicted to have only one unique theoretical peptide, and this was the case for the majority of Alt-ORFs and Novel-ORFs (Supplemental Fig. 6C). Interestingly the agreement between the two proteomics screening approaches was much stronger for those proteins detected with two or more peptides (Supplemental Fig. 6B). When ORFs were identified by a single peptide, the peptide was almost always present in just one of the two studies. Note that ORF detection by single peptide matches, similarly to transcript detection by single read counts, falls into the area of unreliable protein identification, and therefore false discovery in these cases is not controlled (Deutsch et al. 2016). Combined with the fact that many of the alternative isoforms could only be discriminated by a single peptide, the result confirms that the lower number of discriminating peptides in Alt and Novel ORFs versus their PI ORF counterparts impairs their detection by proteogenomics approaches.

Alt/Novel ORFs had lower unique peptide detection rates across all unique peptide ranges (Supplemental Fig. 6C), so other factors are also contributing. To understand whether expression levels were playing a role, we evaluated the number of studies (PSM counts) supporting each ORF to find that, on average, Alt- and Novel-ORFs had five to six supporting studies (median = 2) per detected unique peptide, while this number was nearly 10 for PI-ORFs (median = 4.5), which is in agreement with the notion that PI-ORFs are ubiquitously expressed across tissues (Rodriguez et al. 2013). We found that PI-ORFs detected by unique peptides in fewer than five proteomics studies had a significantly lower expression in our system than those found in more than 10 projects and had similar expression levels as the transcripts coding for Alt- and Novel-ORFs (Supplemental Fig. 6D). Altogether, our results indicate that direct detection in public proteomics databases of predicted coding products of novel and alternative transcripts is hampered by their lower expression pattern and an overall lower identifiability by unique peptides.

Finally, we evaluated the types of protein differences between alternative and principal isoforms for which peptide support was conclusive (minimum of two PSM counts per ORF, *n* = 59 Alt-ORFs, and *n* = 14 Novel-ORFs) and compared them to the composition of our predicted transcriptomes. While our set of curated transcripts predicted that most alternative and novel ORFs distributed between N-terminal truncations, microexons (indels/substitutions up to 9 amino acids [aa]), and major changes (indels/substitutions of more than 9 aa with or without N-Ter/C-Ter truncations), the proteogenomics analysis, as expected, failed to reveal these N-terminal differences and mostly found the major changes both for Alt- and Novel-ORFs (Fig. 5B,C), which is in agreement with a detection approach that relies on positive detection of unique peptides. Microexons were found mostly in Alt-ORFs (Fig. 5B), while Novel-ORFs with no overlap to their PIs were found in the proteomics databases more than expected (Fig. 5C); however, this finding is supported by just a few ORFs and hence cannot be conclusive. Although there was more than a 10-fold difference between the number of identifiable ORFs and those consistently identified in our proteomics screenings, there was a general agreement between the relative abundance of each type of protein difference among the two ORF sets, which suggests that the ORFs confidently identified by unique peptide matches could represent the actual diversity range of the alternative proteome.

### Novel transcripts have a major impact on accurate transcriptome quantification by short reads

Previous studies have shown that the utilization of a reduced, expressed transcriptome as reference for short-read mapping instead of the total reference dramatically impacts transcriptome quantification (Mezlini et al. 2013; Soneson et al. 2016) and improves replicability of expression level estimates (Au et al. 2013). We sought to investigate how the new transcripts impact quantification by short reads. As one important aspect of transcript-resolved analysis is the identification of the transcript that captures most of the expression in each gene (most expressed transcript, MET), we concentrated our study in the comparison of METs when using the total RefSeq (∼160,000 transcripts) or the curated PacBio transcriptome (11,970 transcripts, aka PacBio-T) as reference for short-read mapping. For 3976 genes, the MET was identical in PacBio-T and RefSeq, meaning that the PacBio-T MET was a Full Splice Match of the RefSeq MET. This was not the case for 1433 genes, and, in 996 of them, the PacBio-T MET was a different FSM transcript present in RefSeq. For example, the Signal Peptidase Complex Subunit 2 gene (*Spcs2*) was expressed as one transcript in our PacBio neural transcriptome (PB.6460.1) and had two transcripts in RefSeq quantification (NM_025668 and XM_006508117) (Fig. 5D1). PB.6460.1 is a FSM transcript of NM_025668 and both codify for the PI-ORF of the gene, but the 3′ exon of PB.6460.1 is smaller, resulting in a 3′ UTR shorter by 1340 nt (Fig. 5D1, red dashed box). This shorter 3′ exon is actually the annotated exon of the RefSeq transcript, XM_006508117, which also uses two alternative 5′ exons (Fig. 5D1). XM_ 006508117 was the MET in the RefSeq quantification, while NM_025668 was estimated as poorly expressed (Fig. 5D2). Upon RT-PCR amplification with transcript discriminating primers, we confirmed the PacBio-T- and not the RefSeq-based quantification scheme (Fig. 5D3). When inspecting read coverage at this locus, we observed that neither the unique 5′ junctions of XM_006508117 nor the extra exonic sequence at the 3′exon of NM_025668 were covered by Illumina short reads, while the short-read pattern nicely fits the PacBio transcript model. We speculate that this variability at the 3′ UTRs creates a conflict when resolving transcript quantification in the RefSeq gene model that was decided in favor of transcript XM_006508117 by RSEM (Li and Dewey 2011; Zhang et al. 2017), as this transcript has a more consistent 3′-end coverage. In summary, the transcript quantification error of the *Spcs2* gene when using a reference transcriptome as mapping template was due to a discrepancy in the 3′-end annotation between the reference and the actual expressed transcripts. Similar disagreement patterns were observed for two additional genes, *Dhrs7b* and *Bdkrb2*, with similar outcomes in terms of MET selection (Supplemental Fig. 6E,F). To estimate how general this pattern was, for all the MET discrepant genes, we investigated the RefSeq curation status. The majority of the discrepant genes (57.2%, *n* = 470 genes) corresponded to situations where the PacBio-T MET was a FSM of a manually curated RefSeq transcript and the RefSeq MET was not manually curated, as in the *Spcs2* gene. Furthermore, in these cases, the RefSeq-based MET had significantly worse lowest splice junction coverage and lowest mean exon coverage than the MET called by the PacBio-T quantification (Fig. 5E1,E2). Similarly to *Spcs2*, we found that, for these 470 genes, the differences in the length at the 3′ end between the MET selected at PacBio-T quantification and their matched RefSeq transcripts were significantly higher than in genes where both quantifications selected equivalent METs (Fig. 5E3). Moreover, these differences were also observed for transcripts codifying for the PI-ORF of the genes, indicating that the extensive variability in the 3′ ends that is not annotated in a global reference such as RefSeq is not restricted to secondary/alternative transcripts. These results demonstrate the relevance of using a full-length reference transcriptome updated with novel expressed transcripts for correct quantification estimates.

## Discussion

### SQANTI as a critical tool to analyze whole transcriptome quality

Long-read sequencing technologies, such as the PacBio platforms, as well as Illumina's Moleculo and Oxford Nanopore, have brought novel excitement into the challenge of describing the complexity of the transcriptome of higher eukaryotes by providing new means for sequencing full-length transcript models. While early papers concentrated on demonstrating the dramatic enrichment in full-length transcripts achieved by long reads (Sharon et al. 2013; Tseng and Underwood 2013), there is an increasing number of publications that describe thousands of new transcripts discovered by this technology. Accordingly, we found that, when sequencing the mouse neural transcriptome using PacBio, a large number of novel transcripts could be detected. However, close inspection of these new transcripts revealed signs of potential errors that required a thorough and systematic analysis of these sequences before making any new transcript calls. This motivated the development of SQANTI, a new software for the structural and quality analysis of transcripts obtained by long-read sequencing.

The three basic aspects of the SQANTI QC pipeline are (1) the classification of transcripts according to the comparison of their junctions to a reference annotation in order to dissect the origin of transcript diversity, (2) the computation of a wide range of descriptors to chart transcript characteristics, and (3) the generation of graphs from descriptors data, frequently with a transcript-type breakdown, to facilitate interpretation of the sequencing output and reveal potential biases in the novel sequences. Using this analysis framework, we were able to show that, at least in our mouse experiment, novel transcripts—especially those in the NNC category—are typically poorly expressed transcripts of known genes, consistent with previous reports (Sharon et al. 2013; Tilgner et al. 2014, 2015). We also observed that novel junctions accumulate at the 5′ end of transcripts, have lower coverage by Illumina reads, and are enriched in noncanonical splicing and direct repeats typical of RT switching.

However, none of these features are exclusive of any of the novel transcripts categories, which invites the question on how best to remove transcript artifacts. This has been solved in the past by either eliminating all novel transcripts with at least one junction not supported by short reads (Sharon et al. 2013), by systematically discarding transcripts with noncanonical splicing (Au et al. 2013), or by developing models to estimate the likelihood of a certain splicing event (Abdel-Ghany et al. 2016). In our case, we performed an extensive PCR validation of transcripts belonging to different known and novel types. We found a significant number of transcripts, both with canonical and noncanonical junctions, that had complete junction support by Illumina and that were amplified by RT-PCR of the sequenced cDNA library but that failed to be validated when PCR conditions were adjusted to avoid secondary RNA structures. We concluded that these might be cases of retrotranscription artifacts, which would have escaped filtering solely based on short-read support. This result may suggest that a revision of library preparation protocols is needed, which goes beyond the scope of this study. As an alternative, we were able to combine our set of SQANTI descriptors with a machine learning strategy to build a filter that discards poor quality transcripts with better performance than alternative existing approaches.

The SQANTI filter is data-adaptive, and we showed that it can be successfully applied to other long-read transcriptomics data sets. Note that SQANTI is designed to leverage genome annotation data to characterize and filter the long-read transcriptome. Where no genome is available or the assembly is low-quality, reference-guided correction of transcript sequences will be compromised and therefore also the accurate translation into ORFs. If, additionally, the gene content annotation is poor, this will impact SQANTI transcript classification, leading to enrichment in novel isoforms and genes. In these conditions, it might be difficult to define robust FSM positive and NNC-NC negative training sets for the SQANTI classifier: the first set, because of the low number of known transcripts, and the second, because of poor correction of PacBio sequences. Subsampling experiments showed that 150–200 training set transcripts would be sufficient to obtain comparable performance to that in Figure 4B, indicating that the SQANTI filter can be used confidently even when reduced training sets are available. Furthermore, the SQANTI set of quality descriptors will be extremely useful in these cases, as they will provide a comprehensive characterization of the quality of the transcript calls in situations where little additional data is available. Finally, note that SQANTI is agnostic to the sequencing technology that generated the transcripts and simply requires sequences in FASTA format. Hence, the software can accept transcript sequences from other long-read approaches such as Nanopore and Moleculo. Obviously, the results of the quality assessment will vary as a function of the characteristics of the underlying technology.

### Novel insights in transcriptome complexity from single-molecule, full-length transcriptome sequencing

The fundamental advantage of single-molecule, long-read technologies over short reads is their direct detection of full-length isoform diversity and of novel transcripts. The availability of a curated full-length transcriptome data set of our mouse neural tissue allowed us to explore these aspects confidently. We found that genes with novel transcripts are enriched in metabolic processes and specific neural functions related to neurogenesis and oligodendroglial lineage. This is remarkable because both the narrow control of metabolic programming and the expression of genes involved in cell identity are key players in differentiation courses (Cliff and Dalton 2017), and the finding that most novel transcripts concentrate in these categories suggests that important untapped transcript/regulatory diversity could be revealed by long-read sequencing technologies.

We find it interesting that, while most of the transcript diversity is in the form of novel ORFs, an important fraction of the alternative transcripts are UTR variations of the principal isoform transcript. However, alternative transcripts of the same PI rarely show variable UTRs among them, and novel transcripts infrequently extend annotated TSSs and TTSs. This suggests that gene expression regulation by alternative transcripts either controls the expressed protein or the transcript stability, but the interaction of the two might not be as critical. We also show how high variability at transcript ends is a source of quantification errors that can be alleviated when an expressed full-length reference transcriptome is used. Our data suggests that unannotated alternative polyadenylation events are frequent in mammalian genomes, which, in turn, induce incorrect quantification estimates. Full-length sequencing of the expressed transcriptome readily identifies this 3′-end diversity to provide the correct templates for transcript quantification. On the other hand, variability at the 5′ end is still an issue for full-length transcriptome sequencing, as biological variability cannot be unequivocally differentiated from technical artifacts in cDNA library preparation protocols. The SMARTer protocol typically used in PacBio sequencing may not always capture the full extension of the 5′ ends due to transcript degradation or incomplete retrotranscription. This may account for the lack of 5′-end coverage observed in FSM and ISM transcripts. Trapping of the 5′ CAP prior to the synthesis of the secondary cDNA strand has been shown to increase the overlap of the 5′ end without seriously compromising the yield of long reads (Cartolano et al. 2016) and in the future may represent the preferred form of library preparation to study 5′-end diversity.

Finally, we investigated whether the transcriptome diversity found by long-read sequencing was mirrored by proteogenomics data. We concluded that the low expression and identifiability by single peptides of Alt and Novel ORFs hampered their detection by proteomics. Detection of alternative protein isoforms has proved to be difficult, and while some authors claim that limited detection in proteomics databases might indicate low translational or stability rates (Ezkurdia et al. 2015; Tress et al. 2016), other studies identify a significant proportion of alternative exons associated with ribosomes as evidence of active translation (Sterne-Weiler et al. 2013; Weatheritt et al. 2016). While it is not the scope of this work to resolve these issues, we turned our attention to the analysis of protein differences for those cases of confident peptide detection. We found that the distribution of the type of protein differences in the non-PI-ORFs with respect to the main isoforms is similar to the predictions based on the PacBio sequencing data, except for N-terminal truncations that are at a disadvantage in a standard peptide detection approach. Most of detected alternative ORFs showed major peptide changes compared to the PI-ORFs of their respective genes, which could potentially have an impact on functionality of the alternative protein. While a detailed analysis of these functional differences requires further computational and experimental approaches, the results presented in this paper indicate that long-read technologies, provided adequate quality control is applied, are effective tools for describing the isoform-resolved transcriptome and can aid in the study of the biological significance of alternative splicing.

## Methods

### Differentiation of NPCs and OPCs from neonatal mice

Neonatal C57/BL6 mice (4 d old) were sacrificed and neural precursor cells (NPCs) were isolated from the subventricular zone. Neurospheres were obtained by culturing the progenitors in media supplemented with EGF and bFGF and oligodendrocyte precursor cells were derived from them by adding ATRA (All Trans Retinoic Acid) as described in the Supplemental Methods section.

### Transcriptome generation and quantification

Sequenced PacBio subreads were pooled together, and ToFU software was used to obtain nonredundant transcripts. Default parameters were set to obtain Read of Insert, full-length classification of RoIs, and ICE (Iterative Clustering for Error Correction) steps. The Quiver option was turned on to improve consensus accuracy of previously generated ICE clusters by using non-full-Length read information. Generated HQ polished transcripts (>99% accuracy after polishing) were collapsed to eliminate transcript redundancy (5′ differences were not considered when collapsing transcripts). This set of 5′ merged nonredundant transcripts was defined as the ToFU transcriptome. TAPIS was run with default parameters, except for the maximum intron length used by GMAP (version 2016-05-01), which was set to 200,000. Apart from the reference genome, TAPIS requires the input of a transcriptome annotation file, in this case, the RefSeq murine transcriptome. IDP corrects long sequences through the incorporated LSC (Au et al. 2012) module that maps high quality short reads to Iso-Seq long reads using Bowtie 2 (version 2.3.2) (Langmead and Salzberg 2012). The parameters were set to default except for the aligner (GMAP; see command line in Supplemental Methods) and the minimum isoform fraction value to accept a predicted transcript, which was set to 5%. Transcript quantification using short reads was obtained using STAR (Dobin et al. 2013) as the mapper and RSEM (Li and Dewey 2011; Zhang et al. 2017) as the quantification algorithm (parameters available at Supplemental Methods). Expression estimates were obtained as transcript per million (TPM). Long-read quantification was computed as the number of full-length reads of each transcript divided by the total number of FLs of the sample. The most expressed transcript was defined as the transcript of each gene that obtained the highest average TPM value across all the samples. The relative coverage of a splice junction was defined as the sum of all the reads mapped to the junction divided by the sum of the expression of all the transcripts in which it is present.

### Verification of transcripts by reverse transcription PCR

PCR amplification of selected transcripts was performed with both the sequenced full-length cDNA and newly synthesized cDNA from the same RNA extractions. For new cDNA reactions, 1 µg of total RNA was used to synthesize the first-strand cDNA using SuperScript III (Life Technologies) primed with random hexamers in a reaction volume of 20 µL, according to the manufacturer's instructions. Each random hexamer cDNA synthesis reaction was carried out at two temperature conditions: 42°C and 50°C. RT-PCR reactions used 1 µL of sequenced full-length cDNA or 2 µL of random hexamers cDNA, together with Biotools DNA Polymerase (1U/µL) in a reaction volume of 50 µL. Primers were designed to span the predicted splicing event using Primer-BLAST (Supplemental Table 3; http://www.ncbi.nlm.nih.gov/tools/primer-blast; Ye et al. 2012). PCR conditions were 5 min at 94°C, followed by 35 cycles of 30 sec at 94°C, primer-specific annealing temperature for 30 sec, and 72°C for 1 min or 1 min 30 sec, depending on the predicted product size. PCR amplification was monitored on 1.5% agarose gel.

### RT switching prediction

SQANTI contains an algorithm that implements the RT switching (RTS) conditions described in Cocquet et al. (2006), namely, an exon skipping pattern due to a retrotranscription gap caused by secondary structures in expressed transcripts. The algorithm looks at all the junctions for possible RTS (both canonical and noncanonical junctions) and checks for a direct repeat pattern match at defined sequence locations: The pattern at the end of the splice junction's 5′ exon must match the pattern at the 3′ end of the splice junction's intron. There are three parameters that control pattern matching: (1) the minimum number of nt required to match (4–10); (2) the number of nt of wiggle allowed from the ideal pattern location (0–3); and (3) whether to allow for a single mismatch, indels or not. SQANTI uses as default parameters: a minimum of 8-base-long repeat sequences, a maximum wiggle of 1, and no mismatches. FSM transcripts with the highest mean expression in each gene are assumed to serve as templates for RTS and are excluded from the analysis.

### ORF prediction and functional annotation

The GMST algorithm (Tang et al. 2015) was applied to predict ORFs in PacBio transcripts, setting parameters to consider just the direct strand of the cDNA and AUGs as the initial codon. As GeneMarkS-T allows prediction in incomplete transcripts, lack of coverage at the 5′ end caused some truncated ORFs starting in codons other than methionine. In these instances, the ORF started at the first in-frame methionine, shortening the N terminus. GMST was benchmarked as shown in Supplemental Methods. GO annotation of novel transcripts was done by Blast2GO (Conesa et al. 2005) with default parameters and a query-hit overlap requirement of 90% of the hit sequence (Götz et al. 2008). Enrichment analysis was performed with the hypergeometric test of the GOseq (Young et al. 2010) R package.

### Characterization of Alt-ORFs and Novel-ORFs with respect to PI-ORFs and UTR/ORF variability

Microexon definition was restricted to novel amino acid stretches obtained by in-frame indels or substitutions of no more than 27 nt (9 aa), following Irimia et al. (2014). ORFs showing exclusively N-terminal or C-terminal deletions were labeled as N-Ter Deletion or C-Ter Deletion ORFs. ORFs with indels and substitutions greater than 9 aa, combined or not with N-Ter and C-Ter deletions, were labeled as Major Change ORFs. ORFs that could not be aligned against the PI-ORF of their respective genes were deemed as No align ORFs. Two UTRs were considered to be different if they started in different genomic coordinates or if they shared a common start point but had a length difference of more than 30 nt.

### Machine learning classifier of artifacts based on SQANTI features

A machine learning approach was developed to discriminate artifacts from true novel transcripts utilizing SQANTI features. FSM transcripts were used to define the set of positive transcripts, while NNC-noncanonical transcripts were taken as the negative set. By definition, the labeled sets (FSM and NNC-NC) contain only multi-exonic transcripts, and hence the classifier can only be applied to this type of transcripts. From the total set of SQANTI transcript descriptors, 16 variables defined for both novel and known transcripts sequences were selected (Supplemental Table 1). SQANTI transcript descriptors that relate to reference transcripts, structural category classification, and canonical junction status were excluded because either they are irrelevant to the classification or they were used to define the positive and negative transcript sets. Variables with near zero variance or a correlation higher than 0.9 in the labeled sets were removed. The labeled set was divided into a training set (80%) and a test set (20%) and algorithms were run using down-sampling to equilibrate positive and negative sets and 10×10 cross-validation. Several machine learning methods were tested (Adaboost [Schwenk and Bengio 2000], CART [Breiman et al. 1984], Random Forest [Breiman 2001], SVM [Cortes and Vapnik 1995], and Treebag [Loh and Shih 1997]) on the mouse data that employed 7774 FSM, 1100 NNC-NC transcripts, and 14 SQANTI descriptors (RTS_stage and coding variables were excluded in this data set due to low variability). Random Forest (RF) was selected as the best performing approach and run using 500 trees. This RF approach was also applied to the PacBio maize ear (Wang et al. 2016) and human MCF-7 (PacBio) data sets. For all data sets, we assessed the quality of the predictions by ROC analysis and evaluated SQANTI quality descriptor performance on the filtered transcriptome obtained after the application of the classifier to the novel transcripts. For our mouse data set, SQANTI filter performance was also evaluated on the 67 transcripts tested by RT-PCR by computing ROC, F1-score, and FDR values. The *F*-score was calculated as 2*(Specificity*Sensitivity/[Specificity+Sensitivity]). The FDR was calculated as 100*(FP/[TP+FP]). Note that transcripts evaluated by RT-PCR were excluded from the training set used to build the classifier.

### SQANTI pipeline

SQANTI is implemented in Python with calls to R (R Core Team 2016) for statistical analyses and generation of descriptive plots. The SQANTI program has two major functions: *sqanti_qc.py* and *sqanti_filter.py*. The *sqanti_qc.py* function performs different tasks: (1) It corrects transcript sequences based on the provided reference and returns a corrected transcriptome; (2) it compares sequenced transcripts with the current genome annotation to generate gene models and classify transcripts according to splice junctions (a full description of structural classification of isoforms can be found in the Results section); (3) it predicts ORFs using GeneMarkS-T; (4) it runs our algorithm to predict RT switching; and (5) it returns a transcript level and junction level descriptive file. These files contain 33 and 20 fields, respectively, where the three first fields identify the transcript in the reference genome and the remaining fields describe different transcript/junction properties, making a total of 47 SQANTI descriptors (Supplemental Tables 1, 2). *sqanti_ filter.py* uses the SQANTI features output to perform filtering of artifacts by two different approaches. The intra-priming filter option removes transcripts with adenine stretches in the genomic position downstream from their 3′ ends. The machine learning filter trains a Random Forest classifier based on the user's data following the strategy described above. *sqanti_filter.py* returns a curated transcriptome where artifact transcripts are removed. For the mouse, maize (Wang et al. 2016), and MCF-7 (PacBio) data sets, the reference genomes used were mm10, AGPv4, and hg38, respectively.

### Analysis of peptide support

We performed an in silico analysis of the peptide support for the predicted ORFs in our neural transcriptome by analyzing public proteomics databases. A nonredundant database composed of predicted ORFs from our murine transcriptome experiments and all the murine ORFs annotated in Ensembl (v80) was created. These ORFs were subjected to in silico tryptic digestion (Proteogest, complete digestion). Unique peptides were identified, and ORFs with at least one unique peptide of 7 aa or more were annotated as identifiable ORFs. We then used two different approaches to detect experimental Peptide to Spectrum Matches (PSMs) that match unique peptides from our ORFs. The first approach made use of a pipeline built on Pladipus (Verheggen et al. 2016), a platform that allows for distributed and automated execution of bioinformatics-related tasks, and performed an all-tissue search of mouse proteomic studies (*n* = 36). The pipeline consists of pride-asap, a tool designed to automatically extract optimal search parameters, SearchGUI (Vaudel et al. 2011), a tool that manages the execution of several search engines, and PeptideShaker (Vaudel et al. 2015), a tool that allows for the merging of the results produced by the search engines. For this study, X! Tandem (Craig and Beavis 2004), MyriMatch (Tabb et al. 2007), and MS-GF+ (Kim et al. 2008) algorithms were applied. The input spectra were obtained from 36 murine projects in the PRIDE (Martens et al. 2005) database. The second approach was based on the Sequest algorithm (Eng et al. 1994) and screened large-scale mouse proteomics experiments of brain tissue (Sharma et al. 2015) and astrocyte-secreted proteins (Han et al. 2014). A more detailed description of these approaches is available in Supplemental Methods.

### RNA extraction, full-length cDNA library preparation, and sequencing

Total RNA isolation from cultured cells (two biological replicas per cell type) was done with the Nucleospin RNA kit (Macherey-Nagel) obtaining RINs (RNA Integrity Numbers) between 10 and 9.7 for all samples. The synthesis of full-length cDNA was performed with the SMARTer PCR cDNA Synthesis kit (Clontech, version 040114) following PacBio recommendations. The cDNA synthesis protocol used 1 µg of total RNA, 42°C for retrotranscription, and 13 PCR amplification cycles to control for overamplification of small fragments. For each sample, we performed two first-strand cDNA synthesis reactions and nine PCR reactions using 10 µL of first strand cDNA (diluted 1:5 in TE-buffer) to obtain ∼14–16 µg full-length cDNA per sample. Each sample was submitted to the ICBR sequencing facility (University of Florida) for PacBio sequencing (P4-C2 chemistry). Three cDNA fractions were obtained with BluePippin and sequenced on the RSII instrument using two SMRT cells for the 1–2 kb fraction, and three SMRT cells for 2–3 and 3–6 kb fractions, for a total of eight SMRT cells per sample. Additionally, the same samples were sequenced with the Illumina NextSeq instrument using Nextera tagmentation and 2×50 paired-end sequencing.

## Data access

Sequencing data from this study have been submitted to the NCBI Sequence Read Archive (SRA; https://www.ncbi.nlm.nih.gov/sra) under study accession number SRP101446.

## Supplementary Material

Supplemental Material
