# Supplementary material for: SQANTI: extensive characterization of long-read transcript sequences for quality control in full-length transcriptome identification and quantification
Source: Genome Res. 2018 Mar;28(3):396–411. doi: 10.1101/gr.222976.117 (PMC5848618; doi:10.1101/gr.222976.117)
Supplement: Supplemental Material [file supp_28_3_396__index.html]

SQANTI: extensive characterization of long-read transcript sequences for quality control in full-length transcriptome identification and quantification — Supplemental Material 

# SQANTI: extensive characterization of long-read transcript sequences for quality control in full-length transcriptome identification and quantification

## Supplemental Material

- Supplemental\_Figures.docx
- Supplemental\_Methods.docx
- Supplemental\_Tables.xlsx
- Supplemental\_SQANTI\_Source\_Code.zip
