# Supplementary material for: SQANTI: extensive characterization of long-read transcript sequences for quality control in full-length transcriptome identification and quantification
Source: Genome Res. 2018 Mar;28(3):396–411. doi: 10.1101/gr.222976.117 (PMC5848618; doi:10.1101/gr.222976.117)
Supplement: Supplemental Material [file supp_gr.222976.117_Supplemental_Figures.docx]

**SUPPLEMENTARY FIGURES**

**Supplementary Figure 1. SQANTI classification of the corrected transcriptome and benchmark of the ORF predictor. A)** Sequence context for the insertions observed in ToFU transcripts, after correction with Proovreads and for a simulation of random insertions. For every insertion, a 5 nucleotide window centered in the insertion point was analyzed for its nucleotide composition. **B)** Distribution of transcripts in the Corrected Transcriptome among SQANTI categories. **C-E)** Benchmark of the ORF predictor on the mouse data. **C)** Confusion matrixes for FSM, ISM and UTR3 Fragment transcripts. Most of FSM and ISM were predicted as True Positives (Predicted coding when the reference is coding, 93.7% and 86.1%, respectively). For UTR3 Fragment transcripts, a high percentage of False Negatives (predicted non coding when the matched reference is coding, 80.2%) is expected since these transcripts are largely devoid of coding potential as they are almost entirely encompassed by the matched reference UTR3’. **D)** Types of differences between predicted ORFs and matched reference ORFs for True Positive transcripts. **E)** Size of N-Ter deletions for the FSM and ISM transcripts. FSM transcripts presented much smaller N-Ter deletions than ISM transcripts.

**Supplementary Figure 2. Characterization of the corrected transcriptome. A)** Number of exons of the reference transcripts to which FSM, ISM and UTR3 Fragment transcripts match. **B)** Number of exons per transcript across the different SQANTI classes. **C)** Abundance of single transcript and multi-transcript genes within novel genes and annotated genes. **D)** Number of Full Length PacBio RoIs supporting each transcript across SQANTI classes. **E)** Gene expression for annotated genes (n=7,413) or novel genes (n=341). **F)** Total number of Full Length PacBio RoIs for annotated and novel genes. **G)** Gene and **H)** transcript expression for genes that co-express FSM and NNC transcripts (FSM/NNC genes, n=585) and genes expressing only NNC transcripts (NNC genes, n=123). **I)** Percentage of Adenines in a 20nt window of genomic DNA downstream the TTS. All groups were divided in mono exons and multiexon transcripts but for ISM and NIC. For the ISM SQANTI category, multiexon transcripts are subdivided between 5’, 3’ and internal fragments (see SQANTI documentation) to show the enrichment of Adenines rich windows in the 3’ fragment subset. The NIC category is subdivided in monoexons by Intron Retention, combination of known junctions and no combination of known junctions to reveal the enrichment of Adenines rich windows in the mono exon by Intron Retention subset. **J)** Fraction of each SQANTI category represented by intra-priming candidates.

**Supplementary Figure 3. RT-PCR validation of transcripts.** RT-PCRs for the transcripts assayed in this study.

**Supplementary Figure 4. Ranking of SQANTI descriptor according to their relative importance in the classifier.** SQANTI descriptors represented in different colors are shown intercepting the axis of each dataset at their relative importance. In the MCF7 dataset no Full Length (FL) data was available and the features showing the presence of indels (nIndels and nIndelsJunc) bore no discriminative power and were not used.

**Supplementary Figure 5. Validation of SQANTI across different datasets.** SQANTI was used to characterize the output of two different pipelines used to output transcripts from long reads, IDP **A)** and TAPIS **B)**. IDP finds few novel transcripts and yet they are enriched in bad quality features. TAPIS outputs ~ 5 fold more transcripts than ToFU or IDP, most of them belong to the NNC category and are very enriched in bad quality features. **C)** MCF7 PacBio dataset was used to run ToFU and the results were characterized with SQANTI. The enrichment in bad quality features observed for the NNC category compared to the high quality FSM category is eliminated by the application of the SQANTI filter. D**)** Maize ear PacBio dataset was used to run ToFU and the results were characterized with SQANTI. The enrichment in bad quality features observed for the NNC category compared to the high quality FSM category is eliminated by the application of the SQANTI filter. Non-canonical SJ = NNC transcripts with at least one non canonical splice junction, RT-switching = transcripts flagged as RT-switching candidates, No SR coverage in a SJ = transcripts with at least one splice junction without short read support. Fisher statistical tests, FET, * p < 0.05, ** p <0.01, *** p < 0.001. ns = non significant.

**Supplementary Figure 6. Proteogenomics analysis of predicted ORFs in the curated transcriptome. A)** Distribution of the number of unique peptides predicted for PI-ORFs, Alt-ORFs and Novel-ORFs. Peptides were defined by *in silico* trypsin digestion. **B)** Distribution of the number of detected unique peptides for each ORF category. **C)** Rates of successful detection of each ORF type (number of ORF detected with at least one peptide/ number of ORFs predicted to be identifiable) as a function of the number of predicted unique peptides. **D)** Boxplot of the expression level in our mouse neural data of different ORF types. PI-ORFs are separated between those with more than 10 and less than 5 Peptide Spectrum Matches (PSMs) per detected peptide in the proteogenomics analysis. Kruskal-Wallis test, (K-W), *** p < 0.001. **E)** and **F)** Example of 3’UTR variability in a PI-ORF that leads to a quantification error. **E1)** Transcripts associated to gene Dhrs7b gene according to PacBio sequencing (green) and by RSEM quantification using RefSeq (red). The profile of mapping short reads at the Dhrs7b locus is shown in grey. Position of transcript-specific primers are indicated by yellow arrows. The difference in the TTS between is highlighted by the red dashed box, 0 indicates splice junctions lacking any short-read support. **E2)** Average transcript expression levels for both strategies. **E3)** PCR of Dhrs7b transcripts with specific primers: PB.1035.1/ NM_145428 and PB.1035.2/NM_001172112 but not XM_006532869 were amplified. **F1)** Transcripts associated to gene Bdkrb2 gene according to PacBio sequencing (green) and by RSEM quantification using RefSeq (red). **F2)** Average transcript expression levels for both strategies. **F3)** PCR of Bdkrb2 transcripts with specific primers: PB.1709.2/ NM_009747 but not XM_006515442 and XM_011243991 were amplified.
