# Supplementary material for: SQANTI: extensive characterization of long-read transcript sequences for quality control in full-length transcriptome identification and quantification
Source: Genome Res. 2018 Mar;28(3):396–411. doi: 10.1101/gr.222976.117 (PMC5848618; doi:10.1101/gr.222976.117)
Supplement: Supplemental Material [file supp_gr.222976.117_Supplemental_Methods.docx]

SQANTI**: extensive characterization of long read transcript sequences for quality control in full-length transcriptome identification and quantification**

**Supplemental Materials and Methods**

**Subventricular Zone Tissue Isolation and Neural Precursors Cells Culture**

Neonatal c57/BL6 mice (4 days old) were sacrificed under lethal exposition to CO2 and subventricular zone were isolated. The dissected tissue was placed in fresh washing medium (WM: DMEM/F12 supplemented with 100 units/ml penicillin, 100 µg/ml streptomycin, 5 mM Hepes buffer, 0.125% NaHCO3, 0.09% glucose) and cut into 1 mm^3^ pieces. Under sterile conditions, the tissue was allowed to homogenize incubating in WM for 10 min at 37ºC and then disaggregated up to cellular levels. NPCs were cultured as suspension in Growth Medium (GM: DMEM/F12 supplemented with 100 units/ml penicillin, 100 µg/ml streptomycin, L-Glutamine 2 mM, 5 mM Hepes buffer, 0.125% NaHCO3, 0.6% glucose, 0.025 mg/ml insulin, 80 µg/ml Apo transferrin, 16 nM progesterone, 60 µM putrescine, 24 nM sodium selenite, 4µg/ml BSA, heparin 0.7 U/ml, 20 ng/ml epidermal growth factor (EGF) and 20 ng/ml basic fibroblast growth factor 2 (bFGF)). After seven days, the neurospheres were transferred to low-attach plates and the debris and death cells (cleaning) were progressively removed by repeating centrifugation process and maintained at 37°C in an incubator with 100% humidity and 5% O2.

**Differentiation of Oligodendrocyte Precursor Cells**

Differentiation was performed as previously described^1^.  Briefly, NPCs were cultured with glial restriction media (GRM) which consists of DMEM:F12, B27 supplement (Invitrogen, Carlsbad, CA, http://www.invitrogen.com), 25 µg/ml insulin, 6.3 ng/ml progesterone, 10 µg/ml putrescin, 50 ng/ml sodium selenite, 50 µg/ml Holotransferin, 40 ng/ml Tri-iodo-thyroidin, and supplemented with 4 ng/ml basic fibroblast growth factor (bFGF) and 10 ng/ml EGF (Sigma, St. Louis, MO, http://www.sigmaaldrich.com)  for 1 day.  Subsequently, cells were incubated with 20 ng/ml EGF and 10 µM of all-trans retinoic acid (ATRA) for 1 week. ATRA was then removed and cells were exposed to GRM supplemented with 20 ng/ml EGF for 25 days. At day 28, the spheres were plated in Petri dishes coated with 1:30 Matrigel for 1 week and cultured on GRM supplemented with 20 ng/ml EGF, in which they were maintained for up to 35 days.

**Benchmark of GMST**

To benchmark the quality of the GMST ORF prediction we selected the FSM, ISM and UTR3 Fragment subsets, Supplementary Figure 1B. We defined as True Positives (TP) the cases in which GMST predicted an ORF when the matched transcript had an ORF (93.7%, 86.1% and 18.3% for FSM, ISM and Fragment UTR3 respectively). We defined as True Negatives (TN) the cases in which, GMST did not predict an ORF when the reference did not have one (1.8%, 1.6% and 1.5% for FSM, ISM and Fragment UTR3 respectively). We defined as False Positives the cases in which GMST predicted an ORF when the reference did not have one (3.3%, 3.9% and 0% for FSM, ISM and Fragment UTR3 respectively). Lastly, the False Negatives were the cases in which GMST did not predict an ORF when the reference had one (1.2%, 8.4% and 80.2% for FSM, ISM and Fragment UTR3 respectively). The observation of very high TP and very low TN rates for the FSM and ISM subset is in keeping with the enrichment for protein coding transcripts in the whole transcriptome due to the polyA purification step. The high FN ratio observed in the UTR3 Fragment subset argues in favor of the ORF predictor specificity as it is unable to predict ORFs in regions devoid of coding potential (3’ UTRs) of otherwise protein coding transcripts.

To assess the quality of the predicted ORFs we compared the protein sequence of True Positive cases against that of its cognate reference (Supplementary Figure 1C). For the FSM subset the percentages were: 90.5% of ORFs identical to the reference protein, 7.8% showing a shorter N-Terminus, 0.65% showing major indels and 0.5% not aligning against the Matched reference ORF. For the ISM subset the percentages were: 14.8% of ORFs identical to the reference protein, 55% showing a shorter N-Terminus, 21.6% having a shorter C-Terminus, 6.7% major indels and 1.7% not aligning. In the UTR3 Fragment subset 70% of the predicted ORFs presented major indels and 30% could not be aligned against the PI. The few True Positive UTR3 Fragment transcripts had ORFs that presented major protein changes or that did not align against the matched reference ORF indicating enrichment in this category for incomplete retrotranscription/degradation fragments. The size of the N-Ter deletions was compared for FSM and ISM ORFs classified as N-Ter Deletion, reflecting that, when present, the shortening of the N-Terminus was much smaller in FSM ORFs.

**Analysis of Peptide Support: Neural Tissue approach**

Spectra from mouse proteomics studies PXD001250 and PXD000501 were downloaded from Proteomexchange^4^. The experiments analyzed were carried out using oligodendrocytes, astrocytes, microglia, and cortical neurons^5^ and the secretome of astrocytes^6^. Spectra were searched against a concatenated search target decoy database. This included protein sequences annotated by GENCODE/Ensembl^7^ and our Pacbio ORFs along with an identical number of reversed decoy peptides.

Searches were carried out using Sequest^8^ with the following parameters: one missed cleavage; a minimum peptide length of 7, maximum of 144; precursor mass tolerance: 800 ppm; fragment mass tolerance: 0.03 Da; dynamic modifications: Oxidation +15.995 Da (M) and static modifications: Carbamidomethyl + 57.021 Da (C).

To be positively identified from the spectra the peptide spectrum matches (PSM) had to have a false discovery rate below 1%, a Q-value lower than 0.01 and a charge of 3 or less. Across the experiments 435,156 PSM passed the filters; these PSM came from 39,275 peptides. The peptides identified 9,717 of 22,021 GENCODE genes with 2 or more experimental PSM (a further 2,341 genes with just 1 peptide).

In order to account for common contaminants, the cRAP database was appended to the original murine UniProt sequence database. Peptides that did not match to known coding genes in the GENCODE release were also filtered against the UniProt mouse proteome^9^. A peptide level FDR of 0.05 was imposed for identifications. We filtered all peptides unaccounted for by GENCODE that could be explained as UniProt proteins or as single nucleotide variants. While 6,511 PSM mapped to the 14,448 Pacbio transcripts, almost all of the PSM also mapped to GENCODE and/or UniProt proteins. Just 570 PSM (corresponding to 114 peptides) had a unique mapping to Pacbio transcripts.

**Analysis of Peptide Support: All tissue proteogenomics approach**

A query was performed using the PRIDE web service^10^ (consulted on 06/14/2016) to gather all complete murine projects containing tandem mass spectra (n=36 different projects, PXD000011, PXD000018, PXD000073, PXD000124, PXD000152, PXD000210, PXD000218, PXD000311, PXD000349, PXD000461, PXD000488, PXD000543, PXD000558, PXD000625, PXD000729, PXD000762, PXD000781, PXD000859, PXD000878, PXD001020, PXD001063, PXD001081, PXD001135, PXD001788, PXD001870, PXD001901, PXD001905, PXD002071, PXD002101, PXD002643, PXD002644, PXD002944, PXD003015, PXD003183, PXD003466, PXD003938​). All assays within these projects were then locally cashed prior to processing.

The optimal search parameters (for example precursor and fragment level mass tolerances, and potential post translational modifications) and other required metadata for sequence database searches (for example the applied cleaving enzyme) were automatically inferred for each individual assay using a customized version of pride-asap^11^.

Two separate rounds of sequence database searches were conducted on each individual assay using SearchGUI^12^ (version-2.8.6) and PeptideShaker^13^ (version-1.10.4-beta).

In the first case this was done by searching against all known murine proteins and their isoforms as presented UniProt (release 2016_06) (The UniProt Consortium, 2014). In order to account for common contaminants, the cRAP database^14^ was appended to the original murine UniProt sequence database. Secondly, the searches were repeated using the same settings against a sequence database containing likely protein sequences derived from the Pacbio transcripts.

This methodology required the use of a target-decoy strategy in order to estimate a False Discovery Rate (FDR)^15,16^. The target-decoy databases consist of the original sequences (target) concatenated with the reversed sequences (decoy). Only identifications that were validated at 1% local FDR at the Peptide to Spectrum Match (PSM) level and had a confidence of at least 95% were retained. To estimate the number of false identifications in the entire population and to verify the posted criteria are sufficiently stringent, a global FDR was calculated. The global FDR was calculated by sorting the set of PSMs obtained through the aforementioned searches by the confidence level as provided in the PeptideShaker output. The global FDR for each PSM was then estimated using the following formula:

$$FDRglobal=\frac{decoy}{target}$$

This enabled the generation of a list of potential PacBio transcript candidates with confident mass spectrometry based supporting evidence, making use of all available murine spectra in PRIDE.

**GMAP parameters**

gmap -n 0 -t 4 -d m_musculus_mm10 $fasta --min-intronlength=4 -f 2 > xxx".gtf"

**STAR alignment parameters**

The following command line order based in ENCODE recommendations was used to align the short reads.

STAR --runThreadN 8 --genomeDir $genome_dir --readFilesIn $fasta --outSAMunmapped Within  --outFilterType BySJout --outSAMattributes NH HI AS NM MD --outFilterMultimapNmax 20  --outFilterMismatchNmax 999  --outFilterMismatchNoverLmax 0.04  --alignIntronMin 4  --alignIntronMax 1000000  --alignMatesGapMax 1000000  --alignSJoverhangMin 8  --genomeLoad NoSharedMemory  --outSAMtype BAM Unsorted  --outSAMheaderHD \@HD VN:1.4 SO:unsorted

**Rsem quantification**

rsem-calculate-expression -p 8 --star --output-genome-bam --calc-ci --star-output-genome-bam --estimate-rspd --fragment-length-mean 80 --fragment-length-sd 50 $fasta $reference_name $sample_name

**Glossary**

The following abbreviations are extensively used in the paper:

| **Term** | **Explanation** |
| --- | --- |
| FSM | Full Splice Match. PacBio transcripts matching a reference transcript at all splice junctions or PacBio monoexon transcripts matching a monoexon reference |
| ISM | Incomplete Splice Match. PacBio transcripts matching consecutive, but not all, of the splice junctions of a reference transcript or PacBio monoexon transcripts matching a multiexon reference |
| UTR3 Fragment | Subtype of ISM transcripts that are contained by 95% or more in the 3’ UTR of a reference |
| NIC | Novel In Catalog. PacBio novel transcripts that contain new combinations of known Splice Junctions or PacBio novel transcripts that contain novel junctions formed from already annotated donors and acceptors |
| NNC | Novel Not in Catalog. PacBio novel transcripts that contain novel junctions formed from novel donors and/or acceptors |
| Genic intron | PacBio novel transcripts lying entirely within the boundaries of an annotated intron. They are considered in the manuscript novel genes |
| Intergenic | PacBio novel transcripts lying entirely out of the boundaries of any annotated locus. They are considered in the manuscript novel genes. |
| Genic Genomic | PacBio novel transcripts overlapping partially an annotated intron and an annotated exon |
| Fusion Transcripts | PacBio novel transcripts overlapping partially at least two different loci |
| Antisense | PacBio novel transcripts overlapping at least partially an annotated transcript in the contrary strand |
| MET | Most Expressed Transcript of a gene. The transcript with the highest mean expression across samples for a given gene |

**References**

1. Keirstead, H. S. Human Embryonic Stem Cell-Derived Oligodendrocyte Progenitor Cell Transplants Remyelinate and Restore Locomotion after Spinal Cord Injury. *J. Neurosci.* **25,** 4694–4705 (2005).

2. Kuhn, M. Building Predictive Models in R Using the caret Package. *J. Stat. Softw.* **28,** 1–26 (2008).

3. Breiman, L. Random Forests. *Mach. Learn.* **45,** 5–32 (2001).

4. Vizcaíno, J. *et al.* ProteomeXchange provides globally coordinated proteomics data submission and dissemination. *Nat Biotech* **32,** 223–226 (2014).

5. Sharma, K. *et al.* Cell type- and brain region-resolved mouse brain proteome. *Nat. Neurosci.* **18,** 1819–1831 (2015).

6. Han, D., Jin, J., Woo, J., Min, H. & Kim, Y. Proteomic analysis of mouse astrocytes and their secretome by a combination of FASP and StageTip-based, high pH, reversed-phase fractionation. *Proteomics* **14,** 1604–1609 (2014).

7. Harrow, J. *et al.* GENCODE: The reference human genome annotation for the ENCODE project. *Genome Res.* **22,** 1760–1774 (2012).

8. Eng, J. K., McCormack, A. L. & Yates, J. R. An approach to correlate tandem mass spectral data of peptides with amino acid sequences in a protein database. *J Am Soc Mass Spectrom* **5,** 976–989 (1994).

9. The UniProt Consortium. UniProt: a hub for protein information. *Nucleic Acids Res.* **43,** D204-12 (2015).

10. Vizcaíno, J. A. *et al.* 2016 update of the PRIDE database and its related tools. *Nucleic Acids Res.* **44,** D447-56 (2016).

11. Hulstaert, N. *et al.* Pride-asap: automatic fragment ion annotation of identified PRIDE spectra. *J. Proteomics* **95,** 89–92 (2013).

12. Vaudel, M., Barsnes, H., Berven, F. S., Sickmann, A. & Martens, L. SearchGUI: An open-source graphical user interface for simultaneous OMSSA and X!Tandem searches. *Proteomics* **11,** 996–999 (2011).

13. Vaudel, M. *et al.* PeptideShaker enables reanalysis of MS-derived proteomics data sets. *Nat. Biotechnol.* **33,** 22–24 (2015).

14. Mellacheruvu, D. *et al.* The CRAPome: a contaminant repository for affinity purification-mass spectrometry data. *Nat. Methods* **10,** 730–6 (2013).

15. Elias, J. E. & Gygi, S. P. Target-decoy search strategy for increased confidence in large-scale protein identifications by mass spectrometry. *Nat. Methods* **4,** 207–214 (2007).

16. Elias, J. E. & Gygi, S. P. Target-decoy search strategy for mass spectrometry-based proteomics. *Methods Mol. Biol.* **604,** 55–71 (2010).
