# Supplementary figures and images for: SQANTI: extensive characterization of long-read transcript sequences for quality control in full-length transcriptome identification and quantification
Source: Genome Res. 2018 Mar;28(3):396–411. doi: 10.1101/gr.222976.117 (PMC5848618; doi:10.1101/gr.222976.117)

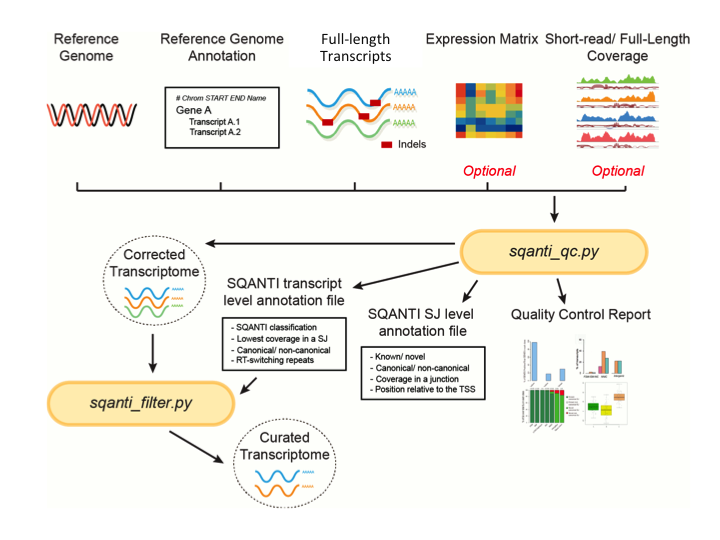

Supplement: Supplemental Material [file supp_gr.222976.117_Supplemental_SQANTI_Source_Code.zip › SQANTI_Source_Code/sqanti_info/sqanti_pipeline.png]
